# Fire spread simulations using Cell2Fire on synthetic and real landscapes

**DOI:** 10.1038/s41598-025-05706-6

**Published:** 2025-07-11

**Authors:** Minho Kim, Cristobal Pais, Marta C. Gonzalez

**Affiliations:** 1 Landscape Architecture and Environmental Planning, University of California, Berkeley, CA 94720 USA; 2 Industrial Engineering and Operations Research, University of California, Berkeley, CA 94720 USA; 3 Civil and Environmental Engineering, University of California, Berkeley, CA 94720 USA; 4 City and Regional Planning, University of California, Berkeley, CA 94720 USA

**Keywords:** Fire spread modeling, Wildfires, Optimization, Natural hazards, Software, Civil engineering

## Abstract

Fire spread models (FSMs) are used to reproduce fire behavior and can simulate fire propagation over landscapes. As wildfires have emerged into a global phenomenon with far-reaching impacts on the natural and built environments, FSM simulations provide crucial information to better understand and predict fire behavior in various landscapes. In this study, we tested Cell2Fire, a recently developed cellular automata-based FSM, against benchmarking models used in the U.S., Canada, and Chile. We experimented on synthetically generated landscapes (homogeneous and heterogeneous mix of fuels), applying Cell2Fire for the first time on U.S. landscapes, and found a high level of agreement between Cell2Fire and existing FSMs. However, FSMs may not always produce realistic simulations. In response, we used two optimization methods to improve the simulation’s accuracy. First, we adopted a multi-objective optimization algorithm that scales the elliptical shape of the Cell2Fire’s output based on rate of spread (ROS) and eccentricity. Second, we optimized four adjustment factors related to the fire spread (head ROS, back ROS, flank ROS, and eccentricity) using blackbox optimization (i.e., derivative-free optimization), minimizing the discrepancy of the output with respect to real burn data. We assessed the effectiveness of the optimization on the 2001 Dogrib Fire in Alberta, Canada and found that the optimized Cell2Fire result more accurately predicted the real burn in comparison to Prometheus (standard Canadian FSM), increasing F1-score from 0.74 to 0.83. Further, Cell2Fire exhibited better computational efficiency, with simulation runtime increasing linearly compared to Prometheus’ runtime increasing exponentially. From these results, users can adjust Cell2Fire and simulate more realistic burns and surpass the capabilities of benchmark FSMs, integrating local or custom-made FSM data to expand the simulator’s application.

**Supplementary Information:**

The online version contains supplementary material available at 10.1038/s41598-025-05706-6.

## Introduction

Climate change has exacerbated fire-conducive weather conditions^1,2^, while urbanization and fuel accumulation from decades of fire exclusion have exposed our communities and environments to a greater risk of catastrophic wildfires^3,4^. The risk of these wildfires poses an immediate and direct impact on our communities, destroying buildings and property, damaging and irrevocably altering ecosystems, and claiming human lives and assets. The far-reaching devastation of wildfires serves as a reminder that we still need to better predict, mitigate, and fight against these hazards. As wildfires continue to occur worldwide^5–7^, there is a pressing need to better understand the potential behavior and effects of fire^8^.

In light of these trends, we explore Cell2Fire^9^ a cellular automata fire growth simulator, which has been applied in Canada^9^, Spain^10^ and Chile^11^. This study presents the first application of the Cell2Fire in the U.S., simulating fire spread across both synthetic and real landscapes. In addition, we adopt an ellipse optimization algorithm^12^ that helps scale the elliptical shape of Cell2Fire’s simulated outputs to resemble those of existing simulators more closely. We also use BlackBox Optimization (BBO)^13^ (i.e., derivative-free optimization) which optimizes adjustment factors for rate of spread (ROS) needed to fine-tune the simulation output with respect to real burn scars.

In fire management, FSMs can be used to simulate and predict wildfire propagation, which helps to better plan and mitigate against wildfire risk^8,14^. These models are often integrated into spatial decision support systems^8,15^ and are essential computational tools for operational wildfire management applications such as burn probability and wildfire risk mapping^9,16–19^, fire suppression^19–21^, landscape management^9,22^, forest tactical management^9,10,16,17^, fuel management^17,23^, prescribed fire allocation^24^ and fire evacuation modeling^25,26^. Semi-empirical FSMs are often used in operational fire management, which are based on simplified abstractions of complex fire physics and use empirically modeled relationships tested with local fuel, topography, and weather conditions^15,27^. However, some semi-empirical FSMs are non-spatial and simulate only a single fuel type under constant input parameters (e.g., Behave^28^ and the Canadian forest Fire Behavior Prediction (FBP) system^29^). To spatially simulate fire spread over heterogeneous landscapes, semi-empirical models can be integrated with a spread simulator to propagate fire growth in a spatially-explicit manner. In particular, FarSite^20^ and Prometheus^30^ are two FSMs that can simulate 2D fire spread used in the U.S. and Canada, respectively, and are considered to be the best-performing in their regions^31^. FarSite uses Behave^28^ and Prometheus uses FBP^29^ to compute fire behavior outputs like rate of spread (ROS). Both FarSite and Prometheus, then, simulate fire growth based on Huygens’ principle of elliptical propagation, which assumes that fire propagates as a wave of independent elliptical wavelets, where every point on the edge of the fire front acts as an independent source of secondary wavelets^32^. This theory has been extended to a set of partial differential equations to model fire growth across a heterogeneous landscape^33^ like in FarSite^20^. Despite their usage in operational wildfire management^20,34^, however, vector-based models can become computationally expensive for larger areas and longer time periods, in addition to causing fire crossovers and unburned islands in the simulations^35^. The computational efficiency of these models scales poorly for large-scale fires and simulations can significantly underestimate real burns. Post-processing would be required to address accuracy issues in the simulations but would require manual adjustments and expert judgment. For instance, FarSite struggles with prolonged fires under changing environmental conditions^36^ and Prometheus relies on adjustments to calibrate to different geographic locations, which further increases computational burden. Alternatively, Cell2Fire was initially developed based on the Canadian FBP system, enabling efficient simulations and the integration of decision-making models^9^. Fire spread in Cell2Fire occurs via cellular automata growth based on the head ROS (HROS), back ROS (BROS), and flank ROS (FROS) computed by the integrated FSM (i.e., FBP). Cell2Fire carries numerous advantages over existing models, including computing efficiency via parallel processing and its modular nature to allow the integration of different FSMs in the processing pipeline. In comparison to Prometheus, Pais et al. (2021) found that Cell2Fire can simulate up to 30 times faster and at a comparable accuracy (above 90% accuracy)^9^.

In this study, we first test Cell2Fire in the U.S. by evaluating against FarSite (i.e., benchmark FSM used in the U.S.) on homogeneous (i.e., single fuel model type) and heterogeneous fuels under constant weather conditions. Testing on heterogeneous landscape shows how well Cell2Fire can simulate spatially explicit fire spread over a diverse set of fuel types. While FSMs can reproduce expected fire behavior, the simulated outputs may not follow the actual propagation of real wildfires. Real wildfires pose multiple challenges including complex fuel mixes, variable weather, and large-scale fire spread, which are relevant to evaluate FSM performance. To address this discrepancy, we use the 2001 Dogrib Fire (Alberta, Canada) as a case study to apply BBO and enhance the accuracy of Cell2Fire simulations with respect to the real burn scar. We selected this case study due to the availability of high-quality, preprocessed datasets, which would be critical for evaluating Cell2Fire and BBO under realistic conditions.

## Results

### Fire spread simulations on homogeneous landscapes

**Fig. 1:**
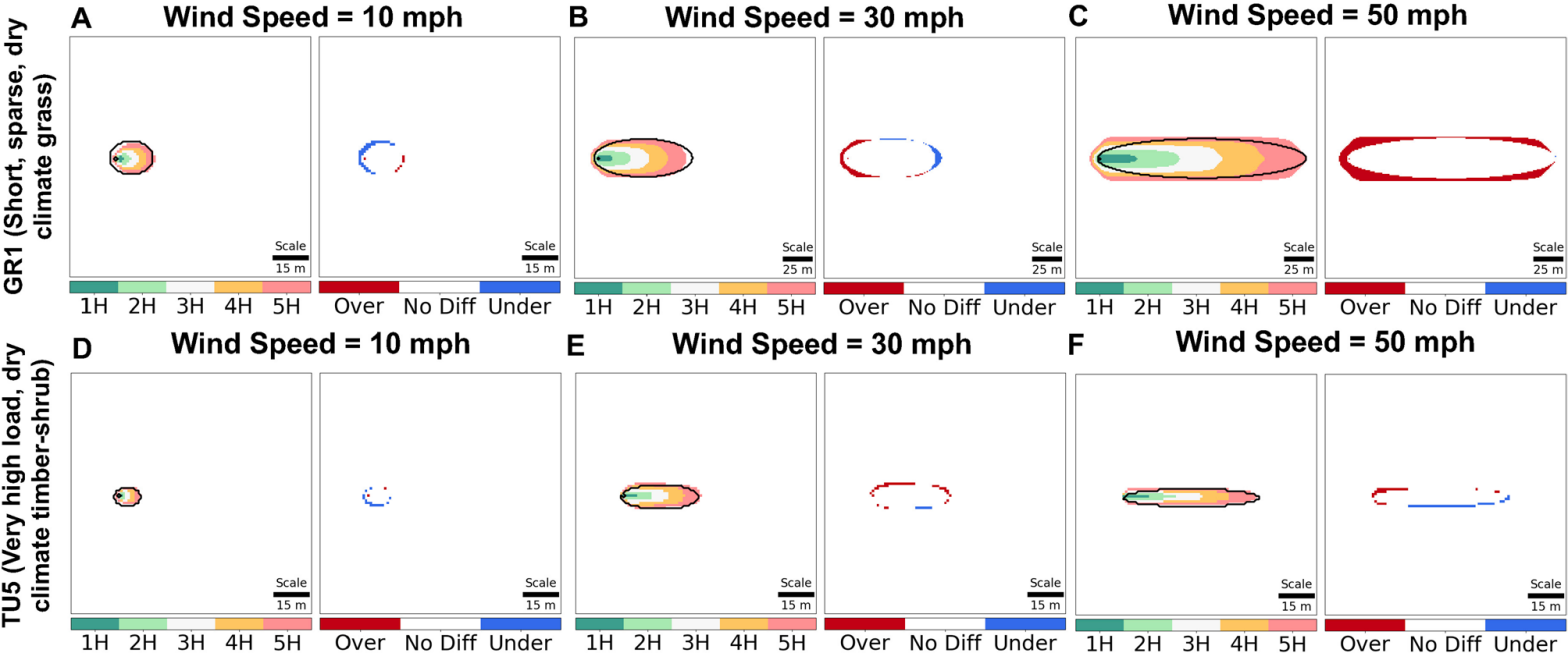
Fire spread simulation outputs on homogeneous landscapes under constant wind speeds comparing Cell2Fire and FarSite simulations. Panels A to C represent simulation outputs for fuel type GR1, while panels D to F for fuel type TU5. For each fuel type, three wind speed scenarios are considered: 10 mph (panels A, D), 30 mph (panels B, E), and 50 mph (panels C, F). The colored regions represent the fire spread simulated by Cell2Fire over five hours. The black-line ellipses represent the corresponding final fire perimeter simulated by FarSite (i.e., perimeter at the 5th hour). The difference maps adjacent to each fire spread simulation illustrate the discrepancy between the final burn area of the two models. Red indicates overestimation by Cell2Fire, blue indicates underestimation, and white indicates no difference.

We simulated fire spread on homogeneous landscapes for different wind speeds over a five-hour duration. We selected the four most common fuel model types found in Northern California (GR1: Short, sparse, dry climate grass; GR2: Low load, dry climate grass primarily grass; GS2: Moderate load dry climate grass-shrub; TU5: Very high load, dry climate timber-shrub). In Fig. 1, we show results from two fuel types (GR1 and TU5) under three different wind speeds (10, 30, 50 mph). The temporal evolution of the fire for Cell2Fire (colored) and FarSite (black outlines) is shown on the left panel, and the amount of under and over-estimation computed as a difference of FarSite and Cell2Fire on the right panel. Cell2Fire’s outputs were similar to those of FarSite at lower wind speeds, as expected due to the low eccentricity and minimal impact of elliptical distortion. At high wind speeds (i.e., 50 mph), however, Cell2Fire tended to overestimate over time in fuels with high expected ROS like GR1. This overestimation trend is also visible in GR2 and, to a lesser extent, GS2 (See Supplementary Fig. S4). This result may be explained by how FarSite can cause severely elongated elliptical shapes due to the high eccentricity at high wind speeds^12,20^.

**Fig. 2:**
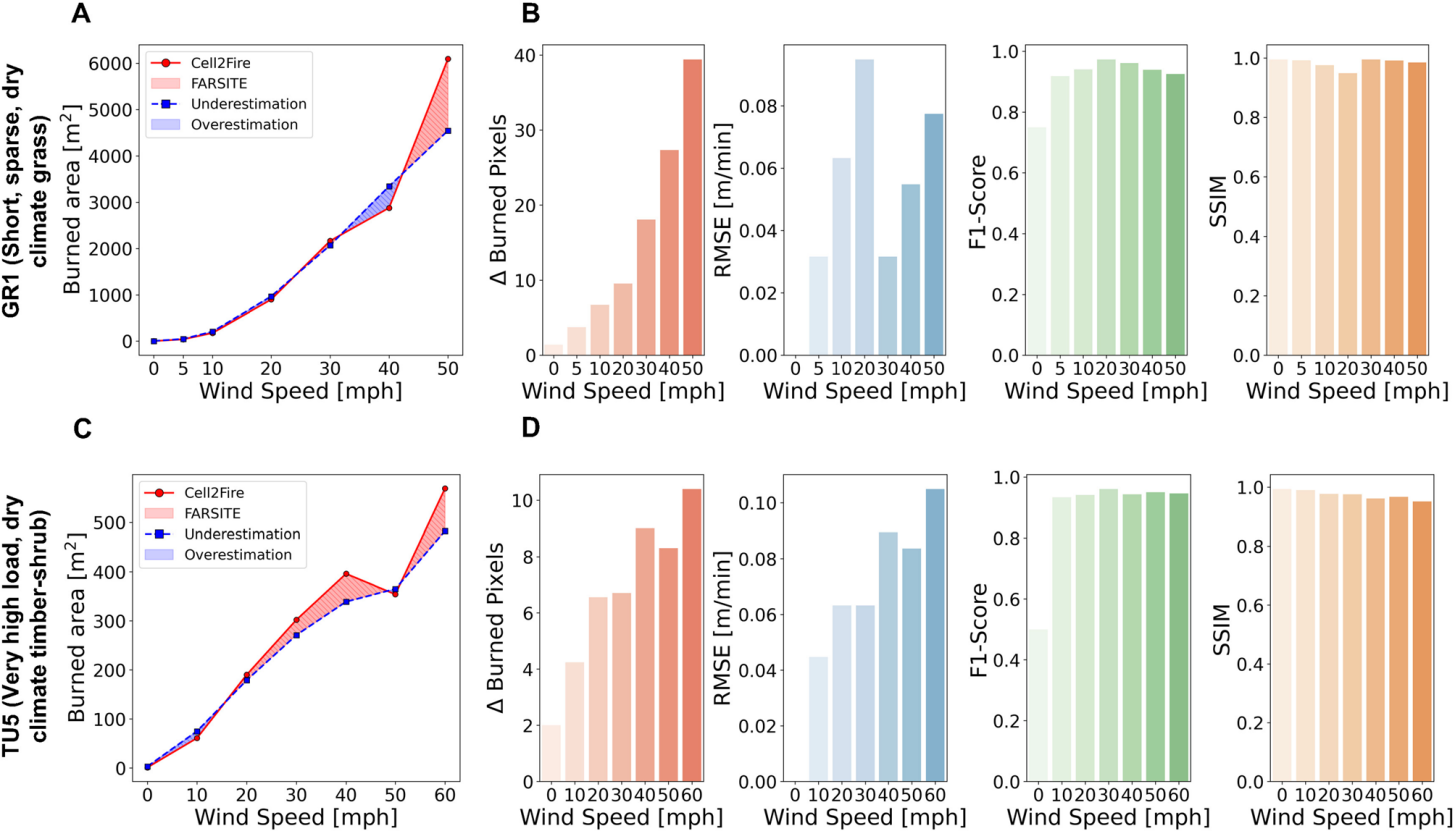
Accuracy and error assessment of fire spread simulations on homogeneous landscapes for fuel types GR1 and TU5. Panels A and B correspond to GR1 while panels C and D correspond to TU5. (**A**) Comparison of burned area in number of pixels over time between Cell2Fire (solid red line) and FarSite (dashed blue line) for GR1. Shaded red regions indicate overestimation by Cell2Fire, and shaded blue regions indicate underestimation. (**B**) Quantitative accuracy metrics for GR1 under varying wind speeds including difference in burned pixels, Root Mean Square Error (RMSE), F1-Score, and Structural Similarity Index (SSIM). (**C**) Comparison of burned area (in number of pixels) over time between Cell2Fire (solid red line) and FarSite (dashed blue line) for TU5. (**D**) Quantitative accuracy metrics for TU5 under varying wind speeds including difference in burned pixels, RMSE, F1-Score, and SSIM.

To better understand the differences in the Cell2Fire and FarSite simulations, the amount of under and overestimation in conjunction with error and accuracy metrics are depicted in Fig. 2 to show the difference between the simulations over different wind speeds, highlighting the amount of Cell2Fire’s underestimation (blue) and overestimation (red). Figure 2B and D display the error (ΔBurned pixels and RMSE) and accuracy metrics (F1-score and structural similarity index (SSIM)) over different wind speeds. In more detail, the accuracy assessment revealed a high level of agreement between Cell2Fire and FarSite as demonstrated by how F1-score and SSIM exceeded 0.9 and 0.95, respectively, for all fuel models and wind speeds. In general, F1-score continuously improved to 30 mph wind speed and plateaued or decreased at higher wind speeds. SSIM decreased slightly with higher wind speeds, while error metrics, RMSE and ΔBurned pixels, reflected this trend and tended to increase.

We also applied Cell2Fire on homogeneous landscapes in Canada and Chile. Fire spread simulation results and error metrics are provided in Supplementary Figs. S5 and S6 for Cell2Fire-FBP results, as well as Table S9 for Cell2Fire-FBP error metrics, while Supplementary Figs. S10-S12 and Supplementary Table S10 show Cell2Fire-KITRAL results and error metrics. For Cell2Fire-FBP, all fuel models returned high F1-scores (F1-score_avg_=0.95) and low RMSE (RMSE_avg_=0.21); however, SSIM was relatively lower (SSIM_avg_=0.78) compared to Cell2Fire-Behave results. This can be explained by the smaller homogeneous landscape grids used for Cell2Fire-FBP, and the simulated fire’s extent would exceed the defined grid. The exception is fuel model D2 (Green Aspen with BUI Thresholding), which yielded a very high SSIM (0.91), thus suggesting that Cell2Fire is capable of highly accurate simulations, regardless of the completion of the fire ellipse. Notably, Cell2Fire-FBP tended to overestimate the back of the ellipse and underestimate the front. For Cell2Fire-KITRAL, the majority of fuel models returned high accuracy and low error (F1-score_avg_=0.91, SSIM_avg_=0.92, RMSE_avg_=0.15). One exception is represented by the PCH1 fuel type (Dense mesomorphic grassland), where simulation led to less accurate results (F1-score = 0.83, SSIM = 0.61). Visualization of the simulation result shows severe underestimation at the back of the ellipse, which is likely due to the rigid shape, rather than being rounded and elliptical. Furthermore, simulations for PCH1 and PCH2 (Sparse mesomorphic grassland) exceeded the grid’s defined size, which can explain the relatively high error and low accuracy results.

## Fire spread simulations on heterogeneous landscapes

**Fig. 3:**
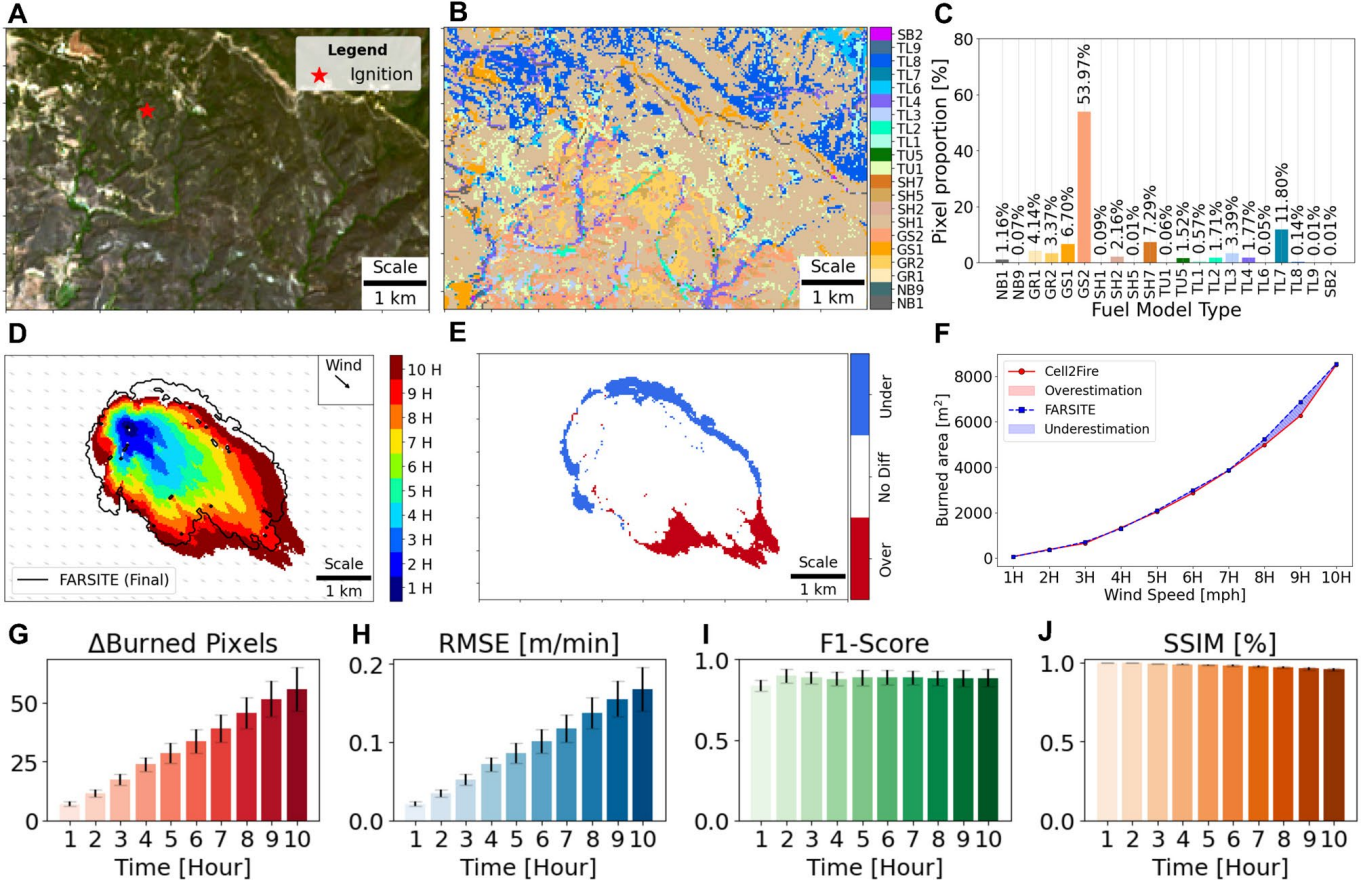
**Fire spread simulation on a real landscape in Santa Barbara county**,** U.S.** (**A**) Landsat-8 satellite image (acquired July 13, 2015) with ignition point. Image downloaded from the Monitoring Trends in Burn Severity platform and plotted using Python libraries Matplotlib (v. 3.5.2) and matplotlib-scalebar (v. 0.8.1). (**B**) Distribution of fuel model types from 2016 LANDFIRE dataset based on the 40-class Scott and Burgan fuel model classification^14^. (**C**) Relative proportion of fuels color-coded by their representation in the fuel map. (**D**) Cell2Fire’s simulation is shown in varying colors to display fire propagation over time, while only the final burn perimeter for FarSite’s simulation is presented by the black-colored perimeter. (**E**) Difference between FarSite and Cell2Fire’s final simulation outputs (after 10 h) showing underestimation at the back of the fire (blue) in contrast and overestimation at the front of the fire (red). (**F**) Comparison of total burn area progression over time. (**G**-**J**) Error (∆ Burned pixels and RMSE) and accuracy (F1-score and SSIM) assessment for burned area over time. Error bars are included based on uncertainty analysis of adjustment factors (HROS, BROS, FROS, eccentricity) for each hour (See Sect. 6 in the Supplementary Materials).

We tested Cell2Fire on heterogeneous landscapes from Santa Barbara, California (U.S.) under constant weather conditions without topography, and Fig. 3 displays the study area and fire spread simulation results with accuracy and error metrics. We selected this landscape because of the numerous fuel types (16 types) including the most common types found in California (GR2, GS2, TU5)^37^ and the heightened risk of wildfires occurring in this region of the U.S. Figure 3A is a Landsat-8 satellite image of the study area. Figure 3B shows the fuel map (30 m spatial resolution) and Fig. 3C plots the spatial distribution and pixel proportion of each fuel model types. Figure 3D displays the fire’s hourly propagation over the 10-hour time period with FarSite’s final fire perimeter (10 h-simulation). The difference between the two fire spread simulation results is shown in Fig. 3E and plotted as a measure of the total burned area over elapsed time in Fig. 3F overestimated the front and underestimated the flank and back of the final fire spread perimeter. The under and overestimation mostly occurred during the final few hours. For quantitative assessment, accuracy and error metrics were plotted and displayed in Figs. 3G-J. F1-score and SSIM were found to consistently be high (*F*1_*average*_ = 0.92 and *SSIM*_*average*_=0.98), while RMSE increased proportionally with elapsed time, thus indicating that Cell2Fire simulations burned a similar area but tended to deviate over time. To enhance the robustness of the simulations, we conducted an uncertainty analysis by running various combinations of adjustment factors (HROS, BROS, FROS, eccentricity) to assess Cell2Fire’s performance. We use refined search bounds for each adjustment factor and find that the simulated result ranges between F1 scores of 0.75 to 0.94 (See Supplementary Fig. S14). We add error bars to Figs. 3G-J for all recorded evaluation metrics. Details on the uncertainty analysis are provided in Sect. 6 of the Supplementary Materials.

## Fire spread simulations on real landscapes

**Fig. 4:**
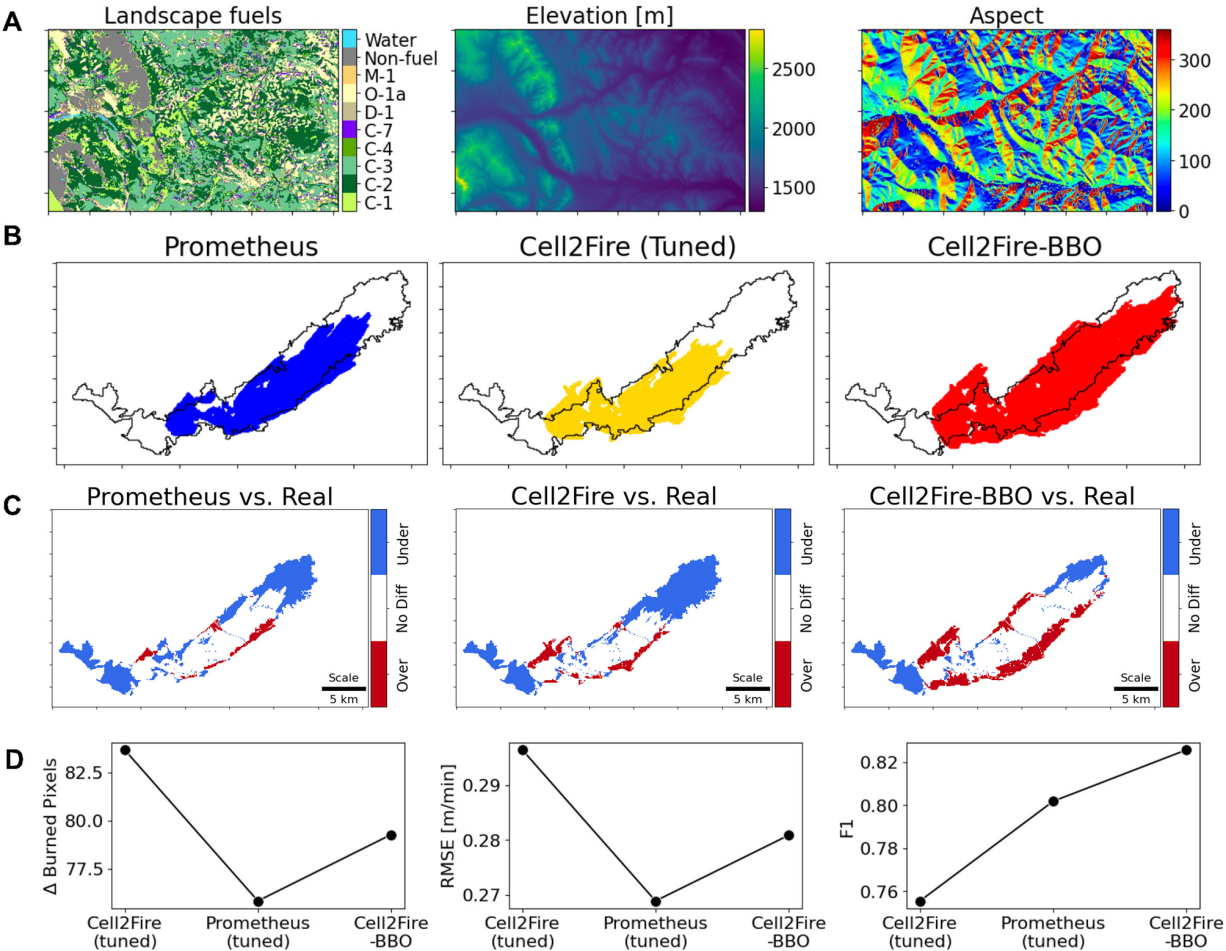
**Fire spread simulation on a real landscape based on the Dogrib Fire (2001)**,** Canada**. (**A**) Spatial distribution of fuel mapped based on FBP along with topographic information (elevation, slope, aspect) from a Digital Elevation Model (DEM). (**B**) Comparison of fire spread simulations from Prometheus, Cell2Fire, and Cell2Fire enhanced using BBO overlaid on the real burn scar (black outline). (**C**) Comparison of Prometheus and Cell2Fire enhanced with BBO, difference between Prometheus and the enhanced Cell2Fire simulations, and the difference between the actual Dogrib fire’s burn perimeter and the enhanced Cell2Fire’s simulation. (**D**) Comparison of error metrics for three FSM instances: Cell2Fire, Prometheus, and Cell2Fire with BBO (Cell2Fire-BBO) versus the real burn scar.

We tested Cell2Fire on real landscapes in west central Alberta (Canada) under real weather conditions from the Dogrib Fire in 2001. We selected the Dogrib Fire because of its detailed fire behavior data and weather stream data provided as an open-source sample dataset with the Prometheus software download^30^. Simulation outputs based on real landscapes in Portezuelo, Chile under variable weather conditions are also provided in Supplementary Section S5. Figure 4A displays the landscape’s FBP fuels, elevation, slope, and aspect. Then, Fig. 4B shows the final fire spread outputs from Prometheus, Cell2Fire, and Cell2Fire optimized with BBO together with the actual burn scar’s perimeter. In Fig. 4C, the differences between each simulation output from Fig. 4B is show in terms of under and overestimation with respect to the actual burn scar. Lastly, in Fig. 4D, accuracy and error metrics were computed for the three instances (Cell2Fire, Prometheus, Cell2Fire with BBO) against the actual burn scar.

Similar to the previous experiments on homogeneous and heterogeneous landscapes, Cell2Fire was able to accurately emulate Prometheus (F1-score = 0.88) with low deviation (RMSE = 0.178 m/min) (See Supplementary Fig. S7A). However, merely mimicking benchmark FSMs is not sufficient to accurately predict the burn scar, as shown by the large underestimation in the instances “Prometheus vs. Real” and “Cell2Fire vs. Real” in Fig. 4C. Comparing Cell2Fire with the real burn scar, the accuracy drops considerably (F1-score = 0.74, RMSE = 0.11 m/min). To address this issue, Cell2Fire was optimized using BBO and the ROS adjustment factors helped improved the simulation’s accuracy considerably with respect to the real burn scar (F1-score = 0.83, RMSE = 0.079 m/min), which is also reflected by a reduction in underestimation in the northeast corner of the burn scar.

We also conducted uncertainty analysis for the Dogrib fire. First, we created 1,000 different weather stream files with randomly perturbed wind speed, relative humidity, and temperature values (perturbation range between 0 and 2). We started with weather parameters^9^ reflecting severe weather conditions in accordance with the Canadian Forest Fire Weather Index (FWI) System (See Sect. 6 in Supplementary Materials). To note, this is the same weather data used for simulations shown in Fig. 4. We discover that F1-scores for Cell2Fire ranges between 0.65 and 0.75, while Cell2Fire with BBO ranges between 0.74 and 0.83 (See Supplementary Fig. S15 in Supplementary Materials).

We also conducted sensitivity analysis to determine the influence of input parameters on Cell2Fire’s simulations (See Sect. 7 in Supplementary Materials). To assess the relationship between adjustment factors and model performance, we analyze the Spearman correlation coefficient on the evaluation metrics for each adjustment factor (HROS, BROS, FROS, eccentricity). We find that eccentricity has the strongest influence on improving accuracy. HROS adjustment is weakly correlated while BROS and FROS have no significant influence. Furthermore, we computed feature importance using Shapley values^38^ on machine learning models trained on BehavePlus data and FBP data (See Supplementary Fig. S16 and Fig. S17, respectively, for SHAP summary plots). We filter the prepared input datasets for the US and Canada and train an XGBoost regression model to predict HROS, BROS, and FROS (See Supplementary Figs. S18 and S19 for train and test loss curves). The SHAP values reveal that wind speed, 1 H moisture content, and 1-hour fuel load was most influential on HROS predictions. For BROS and FROS predictions, 1-hour moisture content, fuel bed depth, and wind speed were the most important. For FBP, the fuel model type and wind speed were the most important features for HROS and FROS predictions. For BROS, fine fuel moisture code (FFMC) and fuel model type were the most important.

## Discussion

In this study, we applied Cell2Fire to simulate fire spread on homogeneous, heterogeneous, and real landscapes in various geographic regions (U.S., Canada, Chile). We generated accurate fire spread simulations comparable to the best-performing FSMs^31^ and enhanced Cell2Fire’s simulated outputs to better reproduce real burn scars via data-driven optimization. In more detail, we optimized Cell2Fire by using BBO and found optimal adjustment factors for HROS, BROS, FROS, and eccentricity to generate more realistic fire spread simulations of historical burn scars, surpassing the capability of conventional benchmark models like Prometheus.

We confirmed that Cell2Fire can emulate benchmark FSMs accurately in homogeneous landscape scenarios. In the heterogeneous landscape scenario, the simulated output tended to be underestimated at the back and flanks as well as overestimated at the front of the burned area. This result can be explained by the homogeneous landscape examples at a wind speed of 10 mph (See Fig. 1), where we can see underestimation at the back and overestimation at the front of the fire ellipse. Nevertheless, we note that Cell2Fire recorded high F1 and SSIM scores, and the over and underestimation regions may appear to be magnified due to the input data’s resolution (30-m resolution) and the figure’s scale. We also confirmed that Cell2Fire was very effective in heterogeneous landscapes as well as even under variable weather and complex terrain in the 2001 Dogrib Fire instance. In particular, the accuracy improvements via optimization in the 2001 Dogrib Fire (F1-score increase from 0.74 to 0.83) reflects a significant reduction in underestimation (false positives and false negatives) with respect to the real burn scar. From a fire management perspective, the consequences of underestimation can be more severe than overestimation. Underpredicting can expose critical areas to heightened levels of uncertainty, thereby escalating risk to life and infrastructure in the unprotected areas. As a result, optimization helps Cell2Fire produce more reliable and realistic predictions to better inform decision-making.

Cell2Fire can help facilitate the development and experimentation of FSMs by fitting the models to updated ROS data and optimizing the simulator with more recent historical burn data. This opens the opportunity for users to use locally created fuel maps and FSM data, providing the flexibility to apply any existing FSMs, experimental FSMs, and even custom rule-based FSMs. To this end, the optimization option can be used as a computational tool for practitioners to find appropriate adjustments as they keep their fire management systems up-to-date by collecting new data such as records of wildfire burn areas. Furthermore, Cell2Fire can be combined with FSMs from different regions to expand the fire spread simulation framework on a global scale. Data democratization together with open-source code such as Cell2Fire help make fire spread simulations more globally applicable and more accessible to a greater diversity of users. For instance, many other countries have also developed their own FSMs such as Australia^39^, South Africa^40^, Portugal^41^ and other countries in Europe^42^. Some FSMs have been developed to work at a global scale^43^. Recent works attempted to map fire behavior data at a global scale, collecting 6,000 individual entries from 33 countries including Australia (25.6%), the U.S. (17.2%), Canada (8.1%), and South Africa (21.1%) as of May 2018^40^. In particular, open-source datasets on cloud-based platforms such as Google Earth Engine can be used as proxy measurements to substitute for complex input features needed to compute FSMs. Future works should exploit these rich data inventories in conjunction with novel, multimodal remote sensing sensors. To note, FSMs can have inherent limitations since semi-empirical models are based on simplified physics (e.g., Rothermel) and are recorded from relatively mild, steady-state environmental conditions^15^. Hence, there is a need to develop more sophisticated FSMs that can reliably reflect extreme fire behavior^8^.

Applying Cell2Fire with BBO is effective for strategic fire management but may be limited for operational real-time applications. For instance, during wildfire operations, data needed for BBO may not be available (e.g., burn perimeter). One alternative option is to find optimal BBO parameters in advance by running simulations on past fires under similar geographic and environmental conditions. While this may help fine-tune Cell2Fire for specific cases, further analysis would be required to adapt Cell2Fire to different ecosystems and wildfire scenarios.

## Methods

**Fig. 5:**
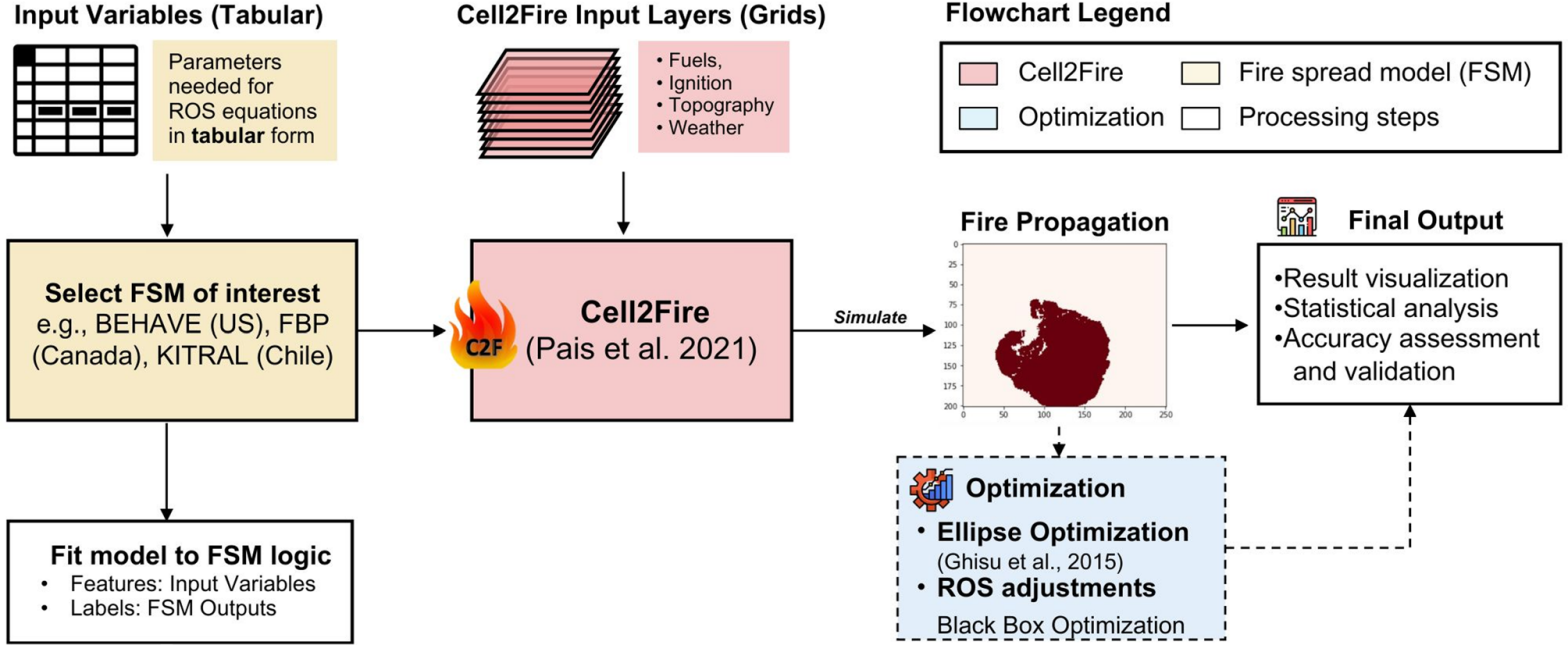
Overview of study’s workflow using Cell2Fire with optimization. Input variables for the FSMs of interest (e.g., Behave, FBP, KITRAL) are used to compute HROS, BROS, FROS. The FSM is integrated in Cell2Fire, where fire growth occurs via cellular automata and FSM logic. The FSM data can also be fitted (e.g., curve fitting, surrogate modeling using machine learning) for sensitivity analysis or ported into Cell2Fire to create custom FSMs. Users can apply ellipse optimization (i.e., multi-objective optimization of elliptical shape factors) to scale simulated outputs with respect to existing simulators like FarSite. Blackbox optimization can also be used to find optimal ROS adjustment factors when a reference shape is available (e.g., real burn scar).

We provide a flowchart of the methodology used in this study in Fig. 5. In the following sections, we explain input datasets used to compute ROS in FSMs, input grids needed to run Cell2Fire, fire growth logic, and optimization techniques.

### Preparing input data for FSMs

We first computed HROS, BROS, and FROS outputs from each FSM (i.e., Behave, FBP, KITRAL). We detail the parameter spaces in Supplementary Tables S6–S8. We then combined the input features and the three ROS outputs in tabular form, such that each row represents data obtained from a single simulated run of the FSM. For Behave, we used the open-source Rothermel package in *R*^44^ to calculate ROS^45^. Input features included the fuel model type, fuel loads (1-hour, 10-hour, 100-hour, woody, herbaceous), moisture of extinction, surface area/volume ratios (1-hour, woody, herbaceous), fuel bed depth, wind speed, wind direction, slope. We also included fuel moisture content based on scenarios (D1L1 (very low dead and live fuel moisture), D2L2 (low dead and live fuel moisture), D3L3 (moderate dead and live fuel moisture), D4L4 (high dead and live fuel moisture)) from Scott and Burgan (2005)^14^. In total, we used 19 input features with varying combinations based on four features (fuel model, wind speed, wind direction, slope, moisture content scenario) to create 218,880 samples (40 fuel models, 19 wind speeds, 4 wind directions, 18 slope values, and 4 moisture content scenarios). Surface ROS using Behave is calculated using the Rothermel equation:

1 $$\:R=\:\frac{{I}_{R}\xi\:(1+{\varphi\:}_{wind}+{\varphi\:}_{slope})}{{\rho\:}_{bulk}\epsilon{Q}_{ig}}$$.

where R is the ROS at the flame’s front, *I*_*R*_ is the reaction intensity, *ϕ*_wind_ is the dimensionless wind factor, *ϕ*_slope_ is the dimensionless slope factor, *ρ*_bulk_ is the bulk density, $\epsilon$ is the effective heating number, and *Q*_ig_ is the heat of pre-ignition^45^. For FBP, input features included fuel model code, wind speed, FFMC, Buildup Index (BUI), and slope factors^29^. Fuel model codes are associated with a set of parameters used to estimate the ROS, which is calculated by a combination of basic ROS derived from experimental results (i.e., extrapolation from field measurements for each fuel type), slope effects, moisture using the FFMC, and accounting for the amount of fuel available for combustion (BUI)^29^. In practice, we varied five main input variables (fuel model, wind speed, FFMC, BUI, and slope), obtaining a total of 180,000 samples (18 fuel models, 16 wind speeds, 5 FFMCs, 5 BUIs, 25 slope values). When computing ROS in the heading direction, the FBP system involves three steps: Calculate ROS using initial spread index and fuel type; modify ROS for slope factor; and modify ROS according to BUI. KITRAL was developed in Chile based on data collected from the previous 30 years. Input features included fuel model type, wind speed, moisture content, heat, fuel load, propagation factor inherent to each fuel, moisture content factor, slope factor, and wind factor. Surface ROS using the KITRAL system is estimated as:

2 $$\:R={F}_{FM}\times\:{F}_{MC}\times\:({F}_{slope}+{F}_{wind})$$

where *R* represents surface ROS; *F*_*FM*_ denotes a fuel model factor for no-wind, no-slope ROS conditions; *F*_*MC*_ corresponds to a moisture content formula where humidity of the fine and dead particles as estimated from relative humidity and ambient temperature; *F*_*slope*_ is a slope factor [%] based on an empirical equation that considers terrain slope spacing; and *F*_*wind*_ is a dimensionless wind factor. In total, 10 input features were used and four features (fuel model, moisture content, wind speed, slope) were varied to generate 110,019 samples (31 fuel models, 21 moisture content scenarios, 13 wind speeds, and 13 slope values).

## Preparing input data for Cell2Fire

For homogeneous landscapes, we selected four of the most common fuel models from the 40-class Scott and Burgan fuels^14^ found in California, namely, GR1, GR2, GS2, TU5^37^. Homogeneous grids for Cell2Fire-Behave were cropped to either 100 by 100 or 500 by 500 pixels – the latter grid size for higher wind speeds and fuels with a higher expected ROS. Homogeneous grids for Cell2Fire-FBP and Cell2Fire-KITRAL were set to 20 by 20 pixels and 80 by 80 pixels, respectively. All homogeneous grids were set to a 100-meter spatial resolution. For the heterogeneous landscape scenario, we used the LANDFIRE 2016 landscape file, which includes a 30-meter resolution fuel map based on the 40-class Scott and Burgan classification^14^. For this study area, we tested constant topographic and weather conditions. We set a constant wind speed of six mph heading southeast and simulated the fire spread for 10 h. Lastly, we set the moisture content to the most extreme weather scenario (“D1L1” scenario in Scott and Burgan (2005)^14^ which corresponds to very low dead fuel moisture with fully cured herbaceous vegetation). For the real landscape scenario, the fuel map was acquired based on the Canadian FBP system at a 100-meter resolution. Topography was set based on the acquired elevation, slope and aspect at the same resolution. Weather stream data was acquired from the Yaha Tinda weather station situated nearby, and the ignition point was set at the coordinate (51.652876, −115.477908) as in Pais et al. (2021b)^9^. The simulation burned for eight hours starting from October 16, 2001, such that the fire spread would capture the major burned area (i.e., 90% of the total burn).

## Integrating FSM logic into Cell2Fire

To run Cell2Fire in different regions, we first fitted ensemble models using input data from an FSM (i.e., Behave, FBP, KITRAL) and integrated the model as a binary executable file into Cell2Fire, where HROS, BROS, and FROS are predicted at each time step for each cell of interest. The input data and ROS outputs can be used to build custom fitted FSMs that can be created in Python and ported into C + + where Cell2Fire runs natively. Cell2Fire intakes regularly-sized grids of fuel, topography (elevation, slope, aspect), and canopy (canopy height, canopy base height, canopy bulk density, canopy cover), as well as a weather stream file defined at regular temporal intervals (wind speed, wind direction). Each cell contains attributes including the fuel type, elevation, slope, location as coordinates, and status for simulation logic (“Available”, “Burning”, “Burned”, “Treated”, or “Non-Fuel”). “Available” cells are burnable, “Burning” cells denote where fire can spread during the time step, “Burned” cells experienced previous burns, “Treated” cells are defined for harvest treatments, and “Non-Fuel” cells are non-burnable and do not ignite. See Supplementary Fig. S1 for a visualization of Cell2Fire’s fire spread process.

- **Ignition**: Given an ignition point (ignition can also be pre-defined point(s) or randomly selected), the coincident cell is ignited, and the fire’s growth may spread to its eight neighboring cells during the time step. In Cell2Fire, this cellular automata growth is modeled as an ellipse emanating from a burning cell to its eight neighbors. The geometry of this ellipse is determined by the HROS, BROS, and FROS, which is then used to compute ROS along each axis to its eight neighboring cells. In more detail, we compute the semi-major axis, semi-minor axis, and eccentricity of the ellipse as a function of ROS (See Sect. 3 Supplementary Materials for a more comprehensive explanation of the elliptical fire spread). We assume that the distance from the centroid of an ignited cell to the centroids of neighboring cells is computed as the straight distance influenced by a slope factor.
- **Fire spread**: Fire spread from a “Burning” cell can reach any of its eight neighboring pixels if the fire reaches the center of the adjacent, available pixel and exceeds a given ROS threshold based on the pixel’s environmental conditions. Once the fire ignites the adjacent pixel(s), the status of the affected pixels is updated (i.e., “Available” to “Burning”), and the fire propagates until the specific ending criterion is reached (e.g., specified time, maximum simulation time, or no more fuel available). Hence, successful fire spread occurs when the ROS along an axis reaches the center of a neighboring cell, thus creating a new ellipse with the neighbor’s cell as the focus.
- **Stopping criteria**: Fire spread ceases when the spread reaches a non-burnable fuel (e.g., Non-Fuel) or the input grid edge and there are no more neighboring cells available, the spread fails to successfully reach neighboring cells, or the simulation time period (defined in the weather stream file) has passed.

### Ellipse optimization and ROS adjustments

Simulated outputs from cellular automata growth model may exhibit an angular elliptical shape in the fire’s heading direction^46–48^ (See Supplementary Fig. S2 for a visual explanation). To emulate existing vector-based FSMs and predict real burn scars more accurately, we adopted a two optimization approaches: (1) Multi-objective optimization using shape correction factors to enhance the ROS distributions at multiple angles^12^ by minimizing the mean squared error and (2) BBO methods^13^ to find optimal parameters of ROS and eccentricity adjustment factors by minimizing the Frobenius norm (i.e., computed between the reference burn and simulated output). Since the optimization algorithm depends on the eccentricity of the simulated output’s elliptical shape (a function of wind speed), the optimization process can be applied universally to any fuel model type and geographic region.

For the ellipse optimization, we used five correction factors designed from ROS-based equations (*K*_1_,., *K*_5_), as proposed by Ghisu et al. (2015)^12^. In more detail, *K*_1_ increases the maximum ROS, *K*_2_ modifies the ellipse’s eccentricity (*E*_*bar*_), *K*_3_ modifies the dependency of ROS on the angle from effective wind direction in the *head* direction, *K*_4_ modifies the dependency of ROS on the angle from effective wind direction in the *back* direction, and *K*_5_ modifies propagation speed in the back direction.

8 $$\:RO{S}^{{\prime\:}}=ROS\:\times\:\:{K}_{1}$$

9 $$\:{E}_{bar}^{{\prime\:}}=\sqrt{1-\frac{1}{L{B}^{{K}_{2}}}\:}$$

10 $$\:tan{\theta\:}^{{\prime\:}}=\frac{tan\theta\:}{L{B}^{{K}_{3}}}$$

11 $$\:tan{\theta\:}^{{\prime\:}}=\frac{tan\theta\:}{L{B}^{{K}_{4}}}$$

12 $$\:RO{S}^{{\prime\:}}\left({\theta\:}^{{\prime\:}}\right)=RO{S}^{{\prime\:}}\left(\frac{1-{E}_{bar}^{{\prime\:}}}{1-{E}_{bar}^{{\prime\:}}\text{cos}\left({\theta\:}^{{\prime\:}}\right)}\right)-\frac{RO{S}^{{\prime\:}}}{1+{E}_{bar}}\left(\frac{1-{E}_{bar}}{1+{E}_{bar}}\right)/{\left(\frac{{\theta\:}^{{\prime\:}}}{\pi\:}\right)}^{{K}_{5}}$$

A multi-objective optimization algorithm was used to optimize these *K* factors and update the elliptical shapes based on eccentricity (a function of wind speed). In this study, we used the open-source *Optuna* hyperparameter optimization package^49^ to find optimal shape correction factors for different eccentricity values, starting from a circle (i.e., no wind conditions where eccentricity equals zero) up to the corner case when the eccentricity of the ellipse approaches a unit value and mimics a thin line in the main wind direction. We optimized the correction factors using mean squared error with the following logic and parameter spaces:

**Table:** 

Minimize	λ_s_
Subject to	1 ≤ *K*_1_ ≤ 2
	1 ≤ *K*_2_ ≤ 2
	0 ≤ *K*_3_ ≤ 1
	0 ≤ *K*_4_ ≤ 1
	1 ≤ *K*_5_ ≤ 2

where *λ*_*s*_ is the shape factor and *K*_*i*_ corresponds to correction factors for *i* = 1 to 5.

For the BBO, we incorporated four adjustment factors for HROS, FROS, BROS, and eccentricity^50^. We initialize the optimization by setting all adjustment factors to a value of 1. Optimal factors are found via a simulator-optimization scheme using BBO^13^ algorithms to test a new set of factors until an $\epsilon$-tolerance equal to 1*e*^−6^ between ten consecutive solutions is achieved or if the algorithm reaches the maximum number of iterations. To obtain robust results, we repeated this process across all fuel types. For the 2001 Dogrib Fire, the best results in terms of the objective function and convergence time of the solution were obtained with the Bound Optimization BY Quadratic Approximation algorithm^51^. We used Frobenius norm as the objective function, which is computed between the real Dogrib Fire’s burn scar and Cell2Fire’s simulated burn scar generated using the updated set of adjustment factors during the optimization. These implementations are available as part of Cell2Fire for future or custom adjustments as needed by researchers and practitioners.

## Electronic supplementary material

Below is the link to the electronic supplementary material.

Supplementary Material 1

## Data Availability

The data that supports the findings of this study are openly available. For the U.S., fuel maps and associated landscape file data are available via LANDFIRE (https://www.landfire.gov). For Canada, the Cell2Fire Github repository provides ready-made samples for simulation, including the Dogrib Fire example shown in this study (https://github.com/cell2fire/Cell2Fire). For Chile, the Cell2Fire-KITRAL adaptation provides ready-made samples for simulation, including the Portezuelo Fire shown in this study (https://github.com/fire2a/C2FK). The simulator used in this study is Cell2Fire, which is available as open-source software. Software Name: Cell2Fire; Developer: David L. Woodruff, Cristobal Pais, Jaime Carrasco; First year available: 2019; Software requirements: Python, C++; Program language: Python, C++; Availability: https://github.com/cell2fire/Cell2Fire. The implementation of this work and sample data are available at: https://github.com/humnetlab/Cell2Fire.

## Declarations

### Supplementary Information

Sections S1 to S7.
