# Supplementary material for: Fire spread simulations using Cell2Fire on synthetic and real landscapes
Source: Sci Rep. 2025 Jul 11;15:25173. doi: 10.1038/s41598-025-05706-6 (PMC12254405; doi:10.1038/s41598-025-05706-6)
Supplement: Supplementary file 1 — Supplementary Material 1 [file 41598_2025_5706_MOESM1_ESM.pdf]

Supplementary Materials

This document contains Supplementary Materials for *"Fire Spread Simulations Using Cell2Fire on Synthetic and Real Landscapes"*. This document contains seven sections (Sections S1 to S7), Tables S1 to S13, and Figures S1 to S19.

Supplementary Table S1. List of abbreviations used in the main text

Category	Abbreviation	Full Form
**Fire spread and behavior**	FSM	Fire Spread Model
	FBP	Fire Behavior Prediction
	ROS	Rate of Spread
	HROS	Head Rate of Spread
	BROS	Back Rate of Spread
	FROS	Flank Rate of Spread
**Inputs**	FFMC	Fine Fuel Moisture Code
	BUI	Build Up Index
	DEM	Digital Elevation Model
**Metrics**	RMSE	Root Mean Squared Error
	SSIM	Structural Similarity Index

1 Fuel model types used in common FSMs

Supplementary Table S2. Fuel model types used in Behave based on Scott & Burgan (2005) (1) (Fuel types 101 to 149). Fuel model types used in homogeneous fuel landscapes are highlighted in bold-text and pinned with an asterisk. Fuel model types that were used in the the real landscape scenario and/or the supplementary materials are highlighted in bold-text.

ID	Code	Description
**101***	**GR1***	**Short, sparse dry climate grass is short, naturally or heavy grazing**
**102**	**GR2**	**Low load, dry climate grass primarily grass with some small amounts of fine, dead fuel**
103	GR3	Low load, very coarse, humid climate grass continuous, coarse humid climate grass
104	GR4	Moderate load, dry climate grass, continuous, dry climate grass, fuelbed depth about 2 ft
105	GR5	Low load, humid climate grass, fuelbed depth is about 1-2 ft
106	GR6	Moderate load, continuous humid climate grass, not so coarse as GR5
107	GR7	High load, continuous dry climate grass, grass is about 3 ft high
108	GR8	High load, very coarse, continuous, humid climate grass
109	GR9	Very high load, dense, tall, humid climate grass, about 6 ft tall
**121**	**GS1**	**Low load, dry climate grass-shrub shrub about 1 foot high, grass load low**
**122**	**GS2**	**Moderate load, dry climate grass-shrub, shrubs are 1-3 ft high, grass load moderate**
123	GS3	Moderate load, humid climate grass-shrub, moderate grass/shrub load
124	GS4	High load, humid climate grass-shrub, heavy grass/shrub load, depth is greater than 2 ft
**141**	**SH1**	**Low load dry climate shrub, woody shrubs and shrub litter, fuelbed depth about 1 foot, may be some grass**
**142**	**SH2**	**Moderate load dry climate shrub, woody shrubs and shrub litter, fuelbed depth about 1 foot, no grass**
143	SH3	Moderate load, humid climate shrub, woody shrubs and shrub litter, possible pine overstory, fuelbed depth 2- 3 ft
144	SH4	Low load, humid climate timber shrub, woody shrubs and shrub litter, low to moderate load, possible pine overstory, fuelbed depth about 3 ft
**145**	**SH5**	**High load, dry climate shrub litter and woody shrubs, heavy load with depth 4-6 ft**
146	SH6	Low load, humid climate shrub, woody shrubs and shrub litter, dense shrubs, little or no herbaceous fuel
**147**	**SH7**	**Very high load, dry climate shrub, woody shrubs and shrub litter, very heavy shrub load, depth 4-6 ft**
148	SH8	High load, humid climate shrub, woody shrubs and shrub litter, dense shrubs, little or no herbaceous fuel, depth about 3 ft
149	SH9	Very high load, humid climate shrub, woody shrubs and shrub litter, dense finely branched shrubs with fine dead fuel, 4-6 ft tall, herbaceous may be present

Supplementary Table S3. Fuel model types used in Behave based on Scott & Burgan (2005) (1) (Fuel model types 161 to 204 and non-burnable fuels 91 to 99). Fuel model types used in homogeneous fuel landscapes are highlighted in bold-text and pinned with an asterisk. Fuel model types that were used in the the real landscape scenario and/or the supplementary materials are highlighted in bold-text.

ID	Code	Description
**161**	**TU1**	**Low load dry climate timber grass shrub, low load of grass and/or shrub with litter**
162	TU2	Moderate load, humid climate timber-shrub, moderate litter load with some shrub
163	TU3	Moderate load, humid climate timber grass shrub, moderate forest litter with some grass and shrub
164	TU4	Dwarf conifer with understory, short conifer trees with grass or moss understory
**165***	**TU5***	**Very high load, dry climate timber shrub, heavy forest litter with shrub or small tree understory**
**ID**	**Code**	**Description**
**181**	**TL1**	**Low load compact conifer litter, compact forest litter, light to moderate load, 1-2 inches deep**
**182**	**TL2**	**Low load broadleaf litter, broadleaf, hardwood litter**
**183**	**TL3**	**Moderate load conifer litter, moderate load conifer litter, light load of coarse fuels**
**184**	**TL4**	**Small downed logs moderate load of fine litter and coarse fuels, small diameter downed logs**
185	TL5	High load conifer litter, light slash or dead fuel, spread rate and flame low
**186**	**TL6**	**Moderate load broadleaf litter**
**187**	**TL7**	**Large downed logs, heavy load forest litter, larger diameter downed logs**
**188**	**TL8**	**Long needle litter, moderate load long needle pine litter, may have small amounts of herbaceous fuel**
**189**	**TL9**	**Very high load broadleaf litter, may be heavy needle drape**
201	SB1	Low load activity fuel, light dead and down activity fuel, fine fuel is 10-20 t/ac, 1-3 inches in diameter, depth < 1 foot
**202**	**SB2**	**Moderate load activity fuel or low load blowdown, 7-12 t/ac, 0-3 inch diameter class, depth about 1 foot, blowdown scattered with many still standing**
203	SB3	High load activity fuel or moderate load blowdown, heavy dead down activity fuel or moderate blowdown, 7- 12 t/ac, 0-.25 inch diameter class, depth > 1 foot, blowdown moderate trees compacted to near the ground
204	SB4	High load blowdown, heavy blowdown fuel, blowdown is total fuelbed not compacted, foliage and fine fuel still attached to blowdown
**91**	**NB1**	**Urban/Developed**
92	NB2	Snow/Ice
93	NB3	Agricultural
98	NB8	Open Water
**99**	**NB9**	**Barren**

Supplementary Table S4. List of the selected fuel model types and their descriptions used in FBP (2). Fuel model types that were used in the the real landscape scenario are highlighted in bold-text.

ID	Code	Description
**1**	**C-1**	**Spruce-Lichen Woodland**
**2**	**C-2**	**Boreal Spruce**
**3**	**C-3**	**Mature Jack or Lodgepole Pine**
**4**	**C-4**	**Immature Jack or Lodgepole Pine**
5	C-5	Red and White Pine
6	C-6	Conifer Plantation
**7**	**C-7**	**Ponderosa Pine - Douglas-Fir**
**11**	**D-1**	**Leafless Aspen**
12	D-2	Green Aspen (with BUI Thresholding)
21	S-1	Jack or Lodgepole Pine Slash
22	S-2	White Spruce - Balsam Slash
23	S-3	Coastal Cedar - Hemlock - Douglas-Fir Slash
**31**	**O-1a**	**Matted Grass**
32	O-1b	Standing Grass
**40**	**M-1**	**Boreal Mixedwood - Leafless**
50	M-2	Boreal Mixedwood - Green
70	M-3	Dead Balsam Fir Mixedwood - Leafless
80	M-4	Dead Balsam Fir Mixedwood - Green
**101**	**Non-fuel**	**Non-fuel**
**102**	**Non-fuel**	**Water**

Supplementary Table S5. List of the selected fuel model types and their descriptions used in KITRAL (3; 4). Fuel model types that were used in the real landscape scenario are highlighted in bold-text.

ID	Code	Description
1	PCH1	Dense mesomorphic grassland
2	PCH2	Sparse mesomorphic grassland
**3**	**PCH3**	**Dense hydromorphic grassland**
**4**	**PCH4**	**Sparse hydromorphic grassland**
**5**	**PCH5**	**Fruit trees, vineyards and orchards**
**6**	**MT01**	**Dense native mesomorphic bushes and shrubs**
7	MT02	Medium to sparse native mesomorphic bushes and shrubs
8	MT03	Dense native hydromorphic bushes and shrubs
9	MT04	Medium to sparse native hydromorphic bushes and shrubs
10	MT05	Formations with predominance of species of the genus Chusquea
11	MT06	Formations with predominance of species of the genus Ulex
12	MT07	Native young stand different from the evergreen forest type
13	MT08	Native young stand of the evergreen forest type
14	BN01	Formations with predominance of Fitzroya cupressoides
15	BN02	Formations with predominance of Araucaria araucana
**16**	**BN03**	**Dense native woodland**
17	BN04	Medium density native woodland
18	BN05	Sparse native woodland
19	PL01	Conifer plantations 0-3 years without management
**20**	**PL02**	**Conifer plantations 4-11 years without management**
21	PL03	Conifer plantations 12-17 years without management
**22**	**PL04**	**Conifer plantations over 17 years without management**
23	PL05	Conifer plantations 4-11 years with management
24	PL06	Conifer plantations 12-17 years with management
25	PL07	Conifer plantations over 17 years with management
26	PL08	New Eucalyptus plantations 0-3 years
**27**	**PL09**	**Eucalyptus plantations 4-10 years**
28	PL010	Eucalyptus plantations over 10 years
29	PL011	Broad-leaf or mixed plantations
30	DX01	Waste from clear-cutting plantations
31	DX02	Waste from clear cutting native forest

2 Comparison of key FSMs and their spread simulators

Some FSMs like FarSite and Prometheus combine a non-spatial FSM (i.e., Behave and FBP) with a spread simulator (US: Behave and FarSite, Canada: FBP and Prometheus, Chile: KITRAL). Supplementary Tables S6–S8 give an overview of key FSMs and their parameter specifications. For more detail, readers are directed to reviews (5; 6; 7).

Supplementary Table S6. Behave input variables, units used in the computation, and the parameter spaces (via the Rothermel R package (8)).

Parameters	Units	Parameter Space
Fuel model	-	40 types
1H fuel load	Mg/ha	Constant
10H fuel load	Mg/ha	Constant
100H fuel load	Mg/ha	Constant
Herbaceous fuel load	Mg/ha	Constant
Woody fuel load	Mg/ha	Constant
Moisture of extinction	%	Constant
1H SAV	m^2/m^3	Constant
Herbaceous SAV	m^2/m^3	Constant
Woody SAV	m^2/m^3	Constant
Characteristic SAV	m^2/m^3	Constant
Fuel bed Depth	cm	Constant
Wind speed	mph	[0, 90], interval=5
Wind direction	Degrees	[0, 270], interval=90
Slope	Degrees	[0, 85], interval=5
Moisture Content	-	4 scenarios based on Scott & Burgan (2005) (1)

Supplementary Table S7. FBP input variables, units used in the computation, and the parameter spaces.

Parameters	Units	Parameter Space
Fuel model	-	18 types
FFMC	-	Constant
Wind Speed	km/h	[0, 75], interval=5
BUI	-	Constant
Slope	Degrees	[0, 100], interval=25
Aspect	Degrees	[0, 180], interval=45

Supplementary Table S8. KITRAL input variables, units used in the computation, and the parameter spaces.

Parameters	Units	Parameter Space
Fuel model	-	31 types
Fuel Load	kg/m^2	Constant
Speed	m/min	interval
Heat	kCal/Kg	Constant
Moisture Content	%	[0, 20], interval=1
Moisture Content Factor	-	Constant
Slope	Degrees	[0, 60], interval=5
Slope Factor	-	interval
Wind Speed	km/h	[0, 60], interval=5
Wind Speed Factor	-	interval

3 Elliptical fire spread in Cell2Fire

Cell2Fire assumes that fires grow elliptically in each burning cell, influenced by the ROS in the head, flank, and back directions (9; 10). HROS is the fire's velocity in the propagating direction aligned with the main axis (0°), while FROS and BROS are the velocities at 90° and 180°, respectively. The ellipse's geometry can be measured by the eccentricity, semi-major axis, and semi-minor axis as a function of ROS over time.

$$a = \frac{HROS + BROS}{2} \times t \quad (1)$$

$$b = \frac{2 \times FROS}{2} \times t \quad (2)$$

$$c = \frac{HROS - BROS}{2} \times t \quad (3)$$

$$e = \frac{c}{a} \quad (4)$$

where a is the length of the semi-major axis, b is the length of the semi-minor axis, c is the distance from the focus to the ellipse center, and e is the eccentricity of the ellipse. The dimensions of these ellipses have been empirically related with wind speed (9; 11) and are assumed to drive the fire's propagation. Higher wind speed can lead to larger eccentricity values, resulting in an elongated shape. This shape can be expressed using the ellipse's length-to-breadth (LB) ratio defined as the ratio between the ellipse's major and minor axes. Different regions and systems have found more suitable LB ratio functions as a function of wind speed (9; 11; 12). Hence, higher wind speeds result in larger LB ratios. The head-to-back (HB) ratio can also be computed from the LB ratio.

$$HB = \frac{LB(WS) + LB(WS)^2 - 1)^{0.5}}{LB(WS) - LB(WS)^2 - 1)^{0.5}} \quad (5)$$

Using these elliptical components with ROS values, we can then express the ROS as a function of wind speed, LB, and HROS:

$$BROS(WS) = \frac{HROS(WS)}{HB(WS)} \quad (6)$$

$$FROS(WS) = \frac{HROS(WS) + BROS(WS)}{LB} \quad (7)$$

Given Cell2Fire's cellular automata nature, the simulated fire can propagate to its adjacent cells in eight directions denoted as an angle θ (i.e., 0°, 45°, 90°, 135°, 180°, 225°, 270°, 315°) (10). Then, these elliptical shape and ROS components can be used to define ROS at each angle and model the fire's propagation (See Supplementary Fig. S1). To aid the explanation of this simulation process, we visually explain this process in three steps. Assuming we have a 3 × 5 grid of cells (all "available"), we start with the ignition at cell i and the fire propagates in the East direction (assuming constant wind speed from the West). All the cells have the same cell size (i.e., regularly-sized grids) and, for simplicity, each cell contains a homogeneous fuel model type. At each time step, all the elliptical components (a, b, c, e) and three ROS values ($HROS, FROS, BROS$) are computed. First, at time=0, ignition occurs at i_6 which changes the cell from "available" to "burning" (highlighted in orange). Next, the ellipse is modeled using HROS, BROS, FROS (determined from the FSM and local fuel, topography, and weather conditions) to output elliptical geometry parameters and compute ROS at each angle using the following expression:

$$ROS(\theta) = \frac{a(1 - e^2)}{1 - ecos(\theta)} \quad (8)$$

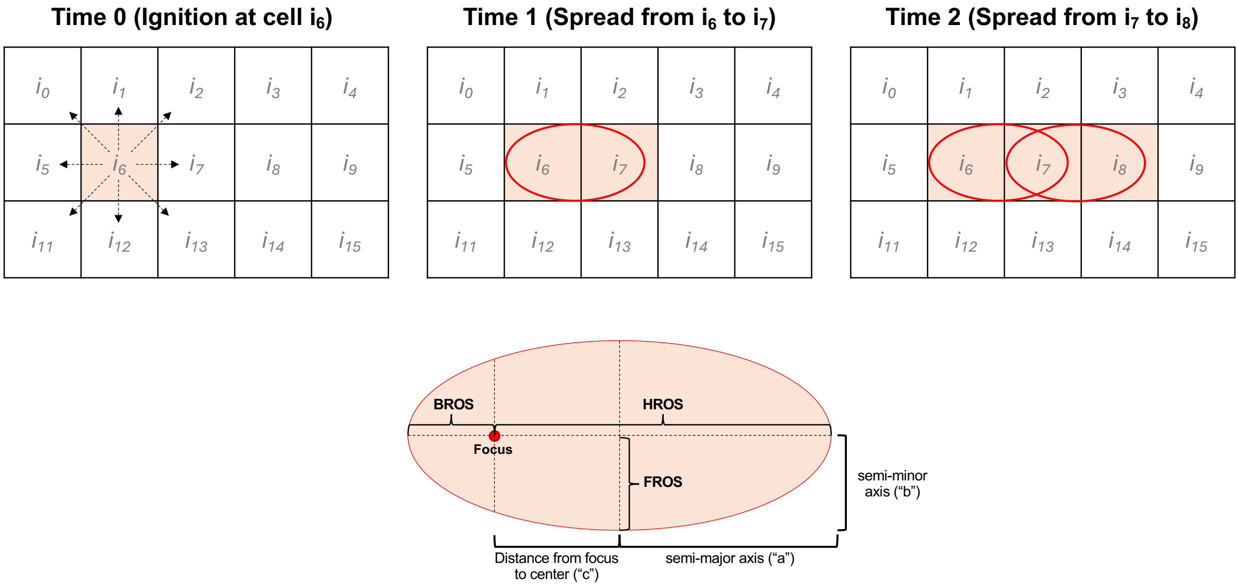

Supplementary Fig. S1. Elliptical fire spread diagram in Cell2Fire, adapted from (10).

We also highlight the importance of ellipse optimization, as outlined in (13). Without optimization, Cell2Fire-Behave tends to underestimate the elliptical shape expected from FarSite. This optimization step is crucial to address the overly elongated shape from high eccentricity (i.e., LB ratio) at high wind speeds as well. We demonstrate the effect of optimization in Supplementary Fig. S2 using the elliptical optimization with shape parameters and ROS adjustments using BBO.

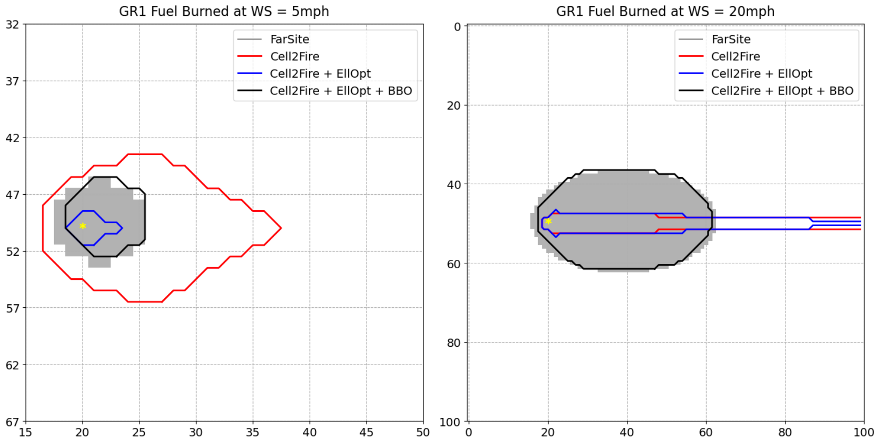

Supplementary Fig. S2. Comparing the effects of Cell2Fire's two-step optimization on elliptical fire growth for different wind speeds on GR1 fuel (Short, sparse, dry climate grass) based on Behave. "EllOpt" refers to simulations using elliptical optimization with shape parameters and "BBO" refers to simulations using BBO adjustments.

3.1 Full homogeneous landscape fire simulations

We visualize the Cell2Fire-Behave and FarSite fire spread simulations over time (5-hour duration) for all wind speeds on homogeneous landscapes of GR1 and TU5 fuels in Fig. S3. We also display the simulations for GR2 and GS2 in Fig. S4, but only show the final burns (i.e., simulation output at five hours).

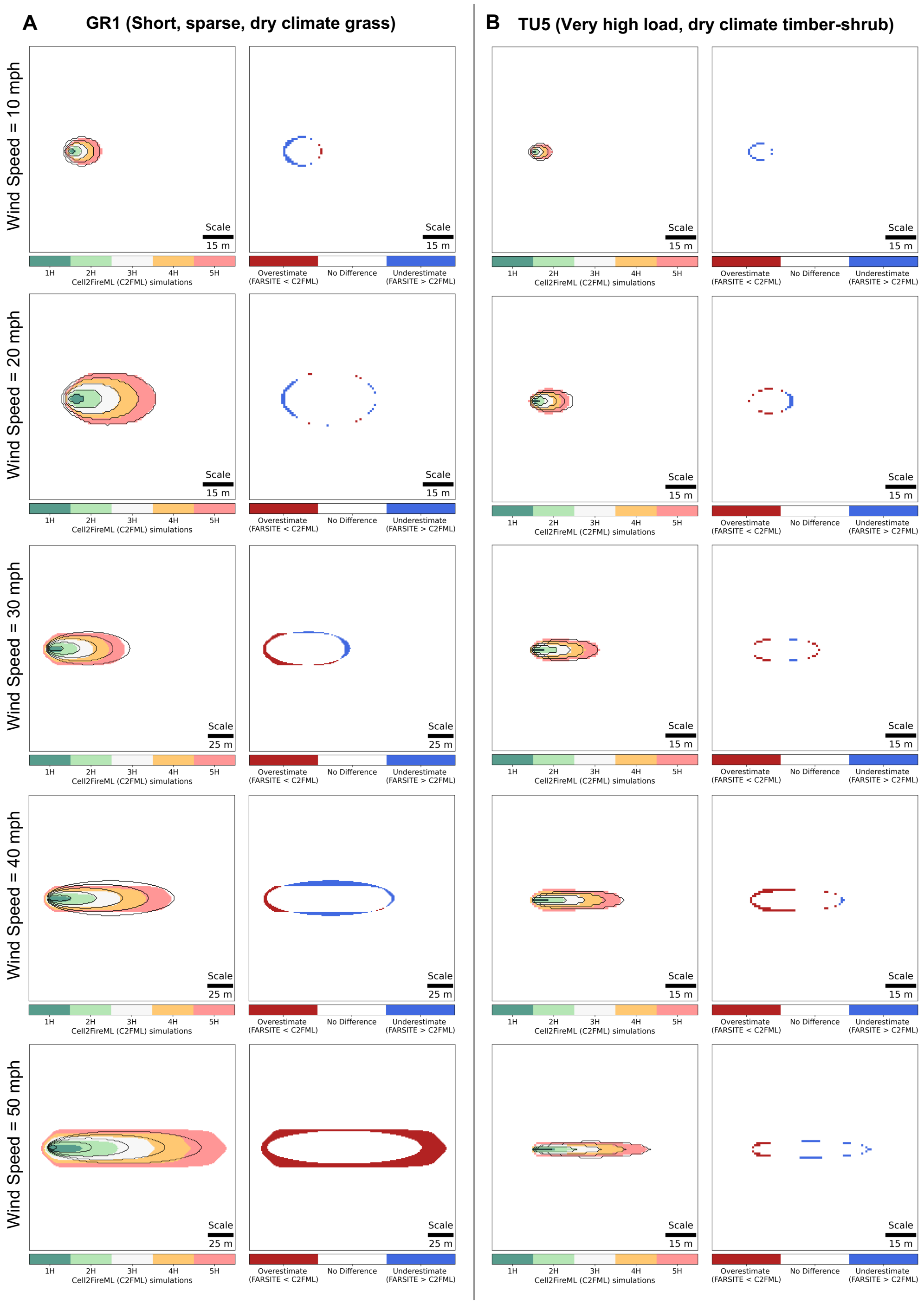

Supplementary Fig. S3. Full comparison of homogeneous fuels for all wind speeds [0, 50] mph for GR1 and TU5 fuels. The varying color scheme shows Cell2Fire-Behave's simulated output over a 5-hour time period, while the black-line ellipses depict FarSite's output. The difference between the two simulation outputs is computed based on the final burn outputs.

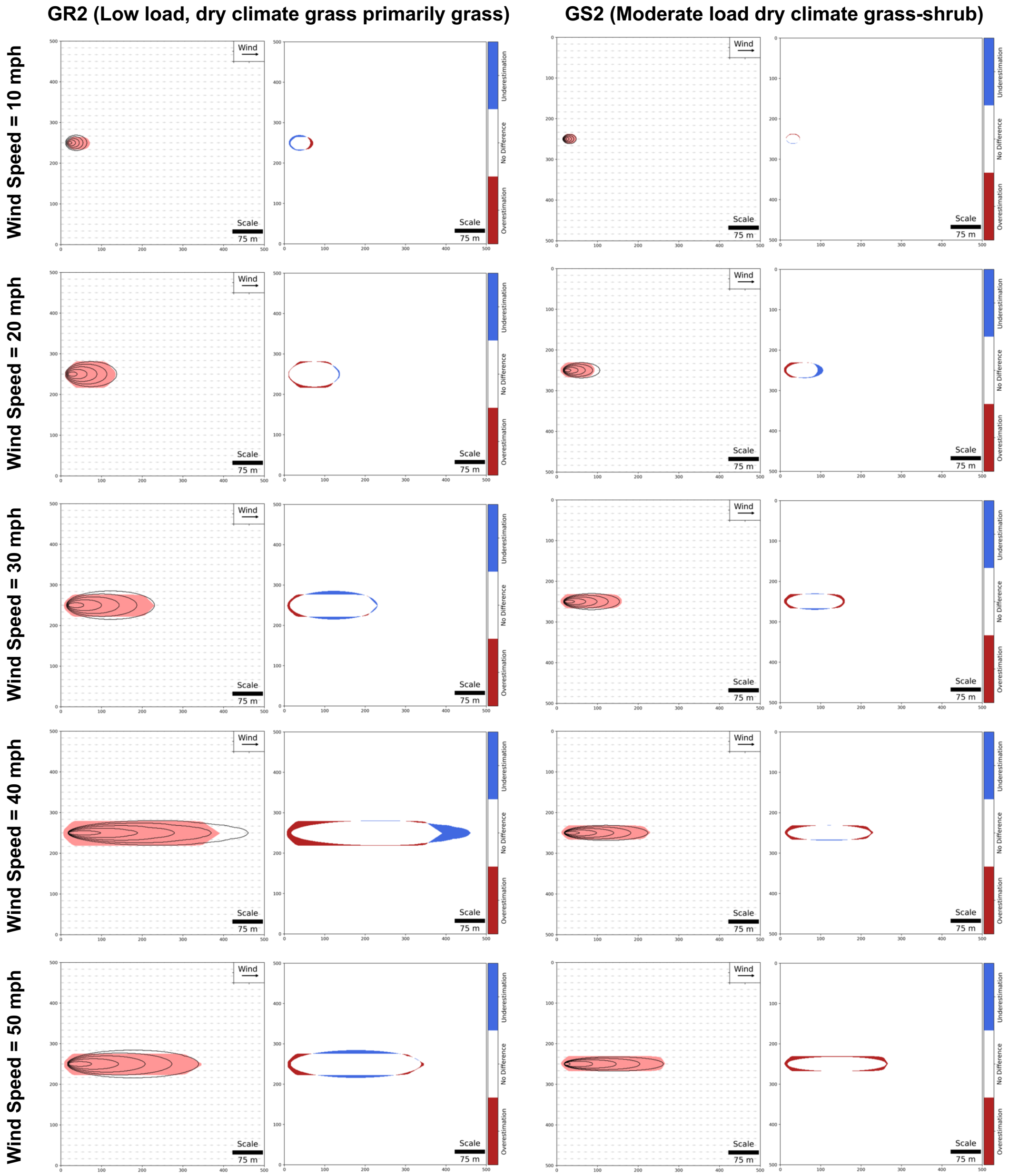

Supplementary Fig. S4. Full comparison of homogeneous fuels for all wind speeds [0, 50] mph for GR2 and GS2 fuels. The colored ellipse shows Cell2Fire-Behave's simulated output at the end of the 5-hour time period, while the black-line ellipses depict FarSite's output. The difference between the two simulation outputs is computed based on the final burn outputs.

4 Cell2Fire simulations in Canada using FBP

We use FBP in Cell2Fire (see Table S9 for accuracy results) and simulated on homogeneous landscapes (Figs. S5 and S6) and a real landscape with the actual wildfire burn scar in Fig. 4 in the main text. We provide error and accuracy metrics to demonstrate how Cell2Fire-FBP can emulate Prometheus simulations successfully.

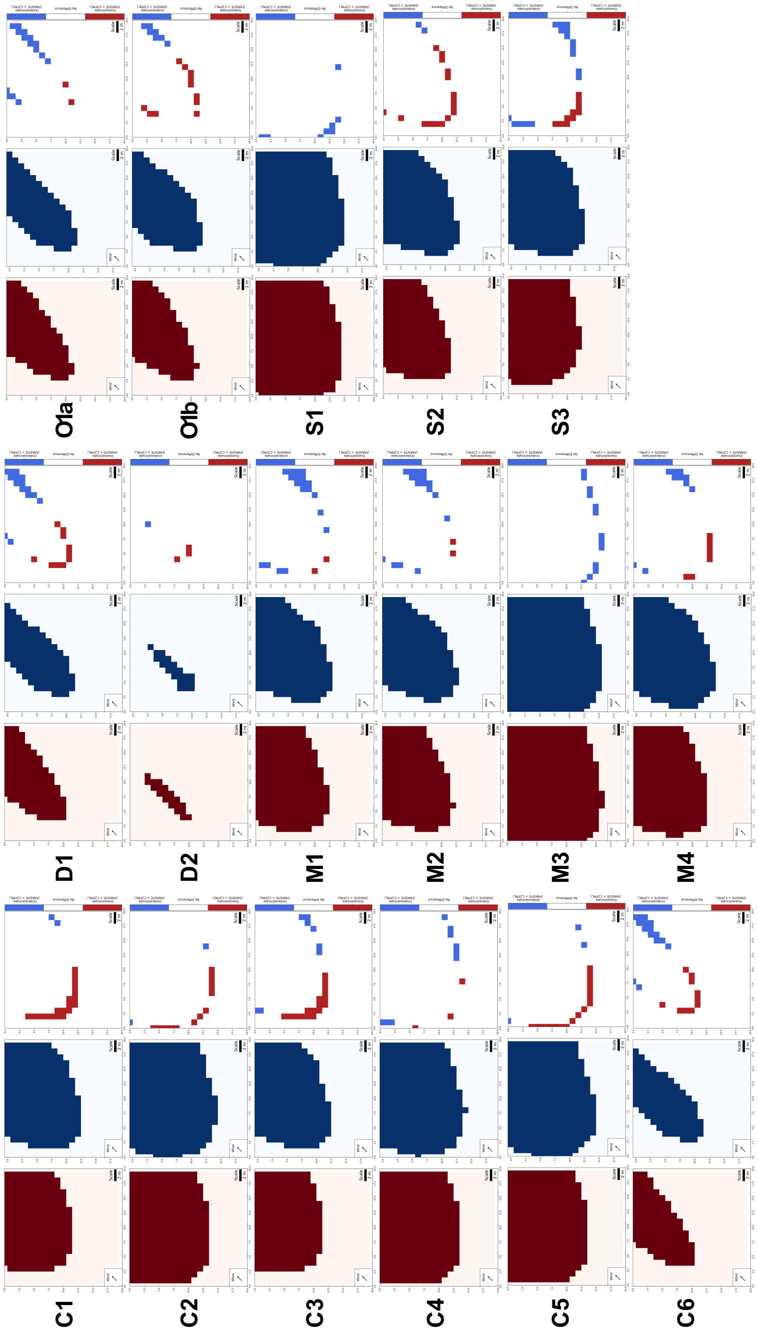

Supplementary Fig. S5. Full comparison of homogeneous fuels in the FBP system (Canada).

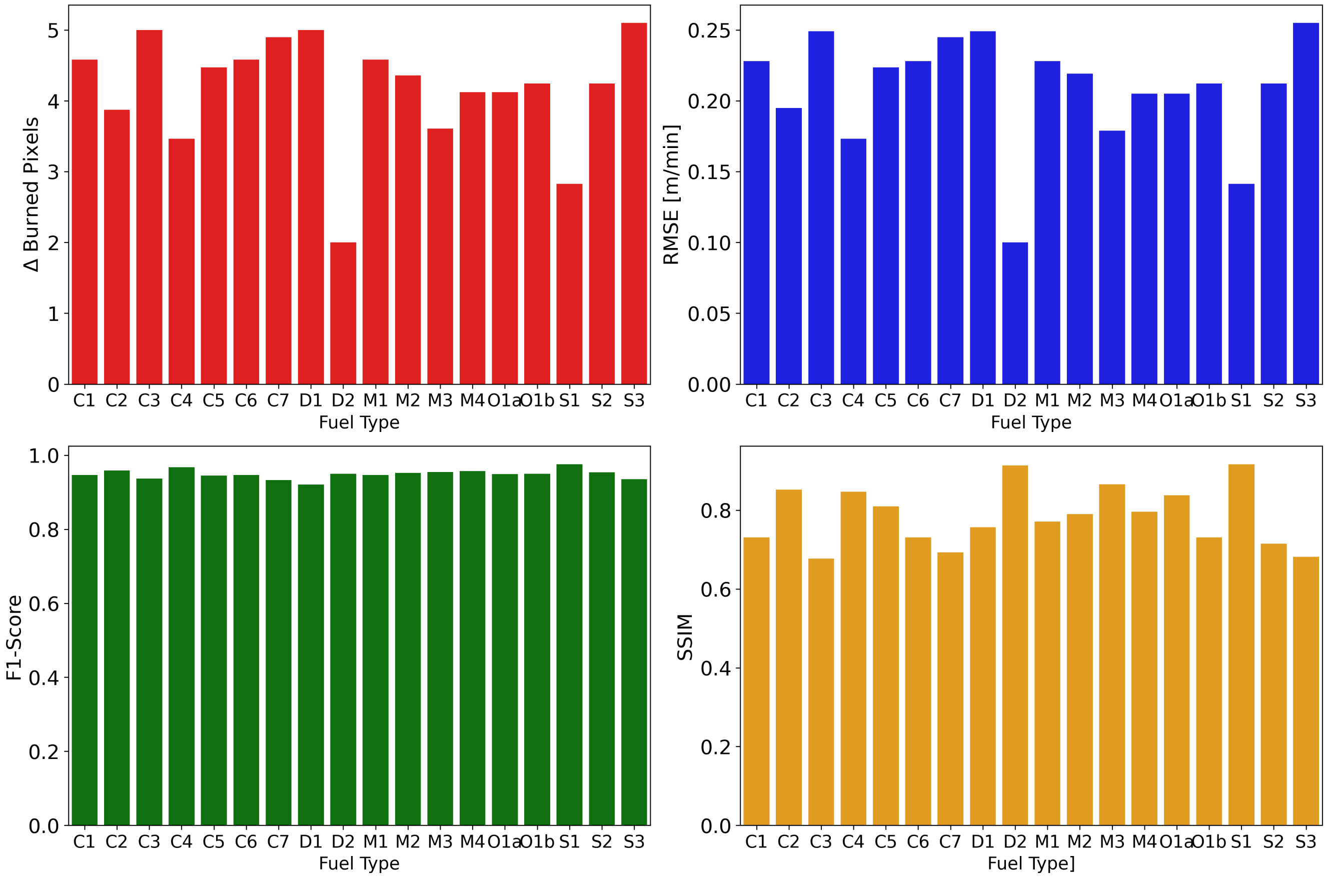

Supplementary Fig. S6. Accuracy and error metric plots for Cell2Fire fit on FBP (Canada).

Supplementary Table S9. Accuracy and error metric results from Cell2Fire fit on FBP (Canada), including computation time of simulation.

Fuel Type	Δ Burned Cells	RMSE [m/min]	F1	SSIM	Time [s]
C1	4.58	0.23	0.95	0.73	0.09
C2	3.87	0.19	0.96	0.85	0.11
C3	5.00	0.25	0.94	0.68	0.11
C4	3.46	0.17	0.97	0.85	0.11
C5	4.47	0.22	0.95	0.81	0.09
C6	4.58	0.23	0.95	0.73	0.09
C7	4.90	0.25	0.93	0.69	0.09
D1	5.00	0.25	0.92	0.76	0.08
D2	2.00	0.10	0.95	0.91	0.04
M1	4.58	0.23	0.95	0.77	0.09
M2	4.36	0.21	0.95	0.79	0.03
M3	3.61	0.18	0.95	0.87	0.09
M4	4.12	0.20	0.96	0.80	0.04
O1a	4.12	0.20	0.95	0.84	0.09
O1b	4.24	0.21	0.95	0.73	0.08
S1	2.83	0.14	0.98	0.92	0.11
S2	4.24	0.21	0.95	0.72	0.11
S3	5.10	0.25	0.94	0.68	0.10
**Average**	**4.17**	**0.21**	**0.95**	**0.78**	**0.09**

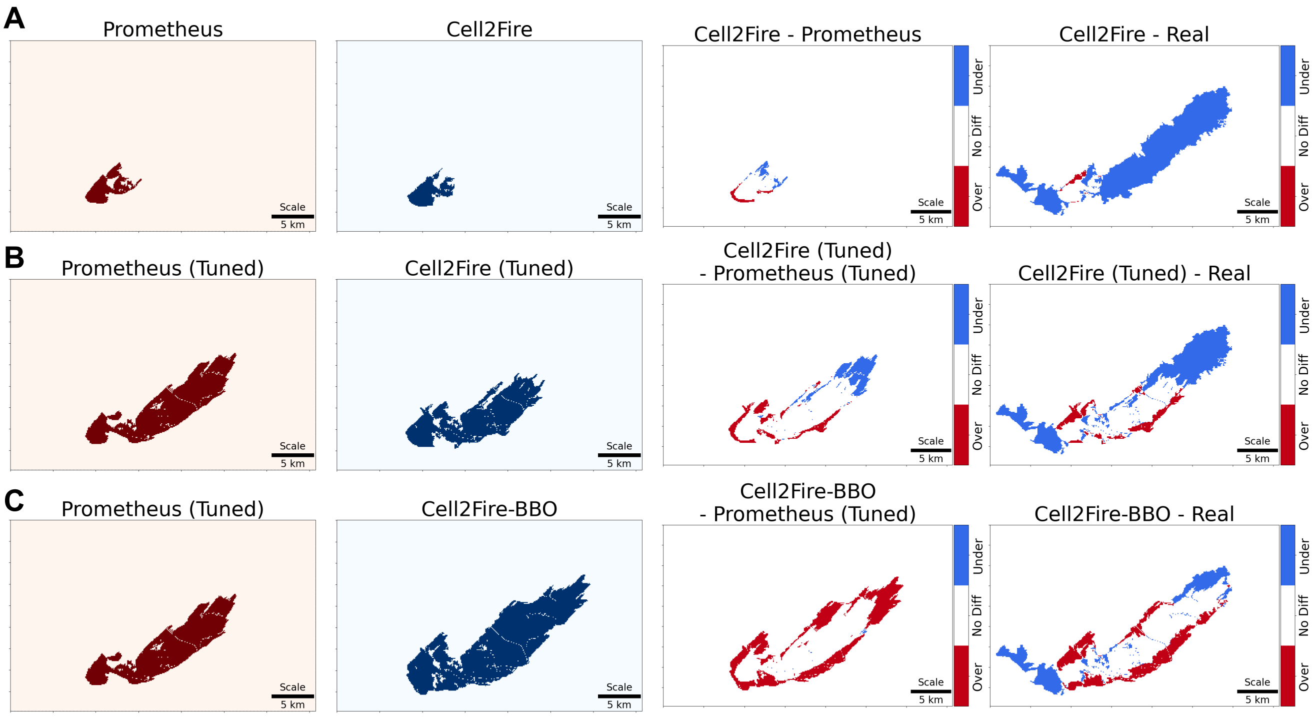

Supplementary Fig. S7. Fire spread simulation on a real landscape in Canada following the Dogrib Fire. (A) Comparison of Prometheus with Cell2Fire and the real burn scar. (B) Comparison of Prometheus with Cell2Fire optimized with BBO and the real burn scar.

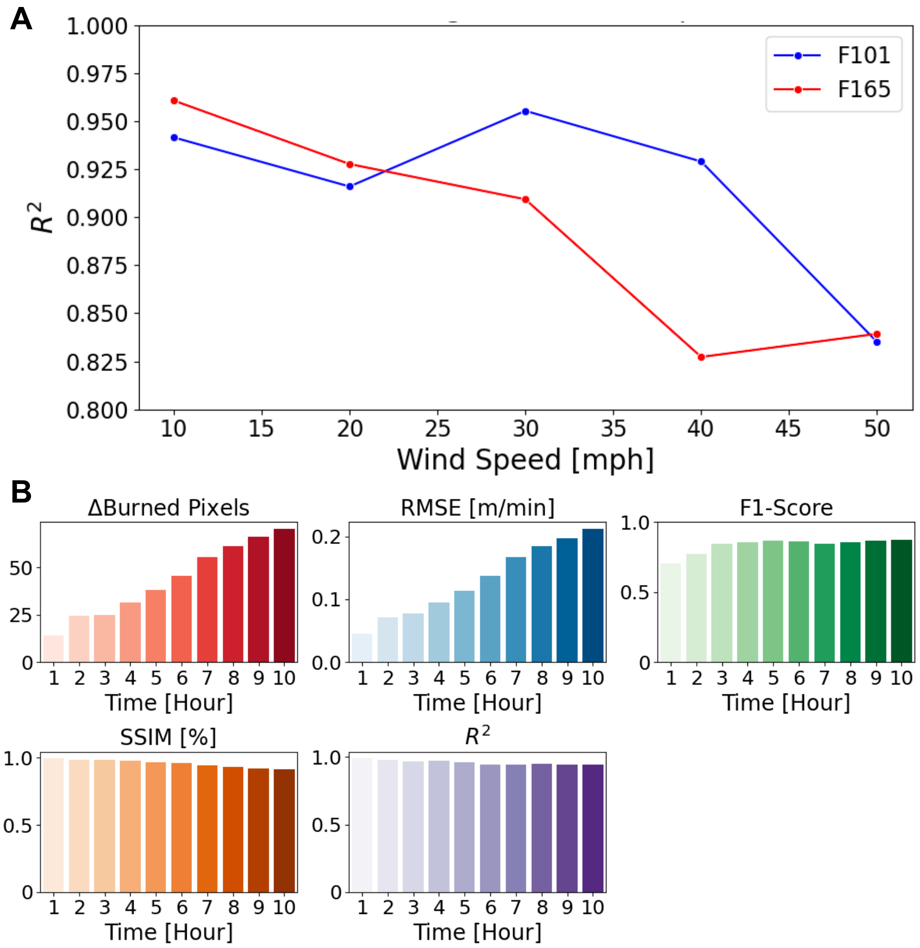

Supplementary Fig. S8. Evaluation metrics of Cell2Fire simulations on homogeneous and heterogeneous landscapes in the U.S. (A) R^2 results on homogeneous landscapes. (B) All evaluation metrics including R^2 results on heterogeneous landscape.

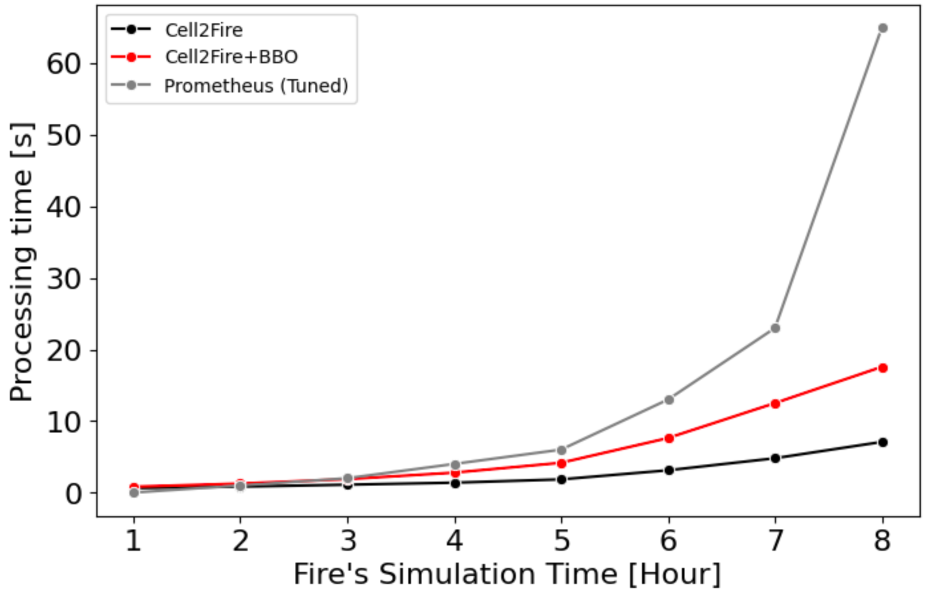

Supplementary Fig. S9. Comparison of computational time for running fire spread simulations.

5 Cell2Fire simulations in Chile using KITRAL

We use KITRAL in Cell2Fire (see Table S10 for accuracy results) and simulated on homogeneous landscapes (Figs. S10–S12) and a real landscape in Portezuelo, Chile (Fig. S13).

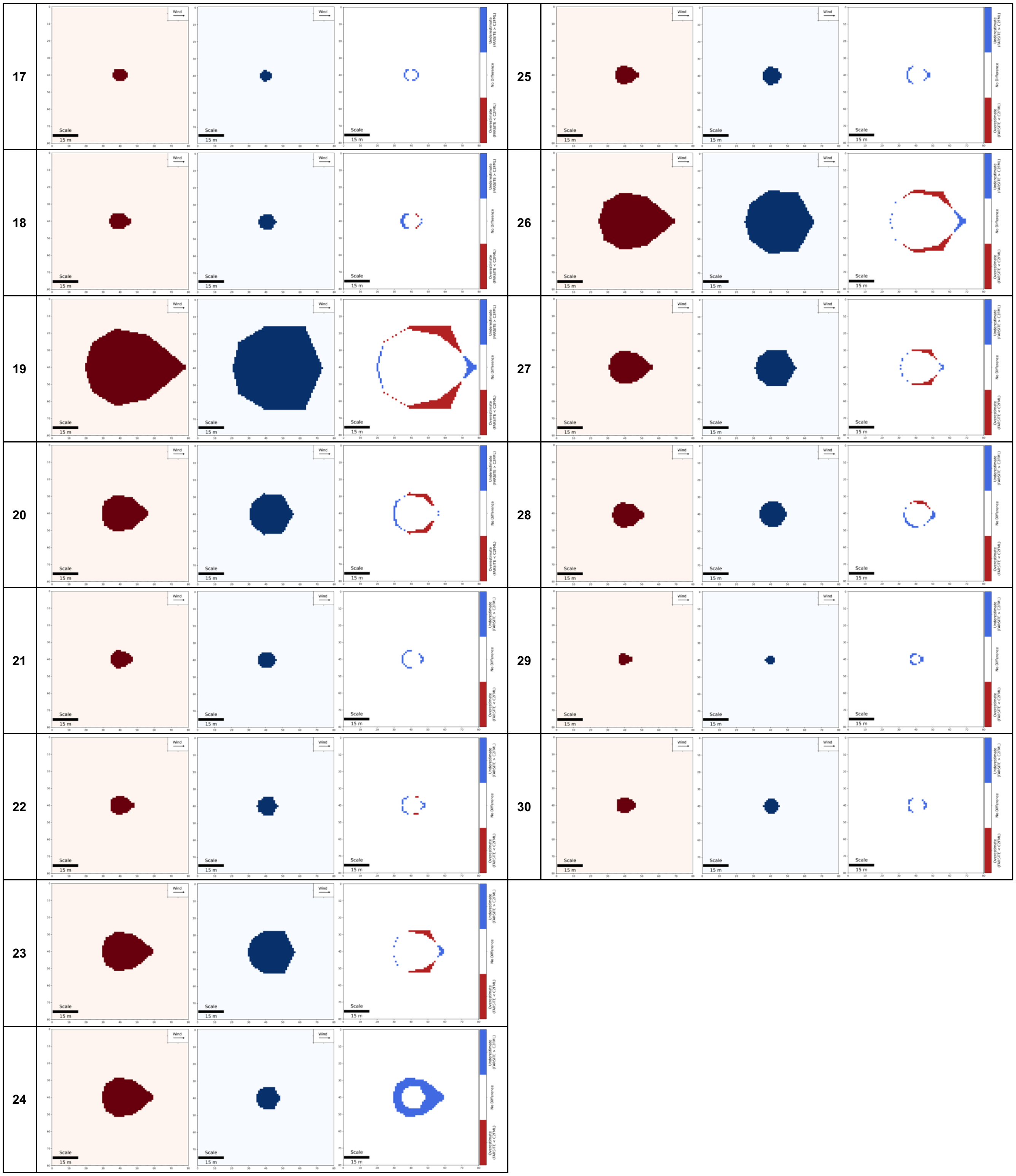

Supplementary Fig. S10. Full comparison of homogeneous fuels in the KITRAL system in order of fuels shown in Table S10.

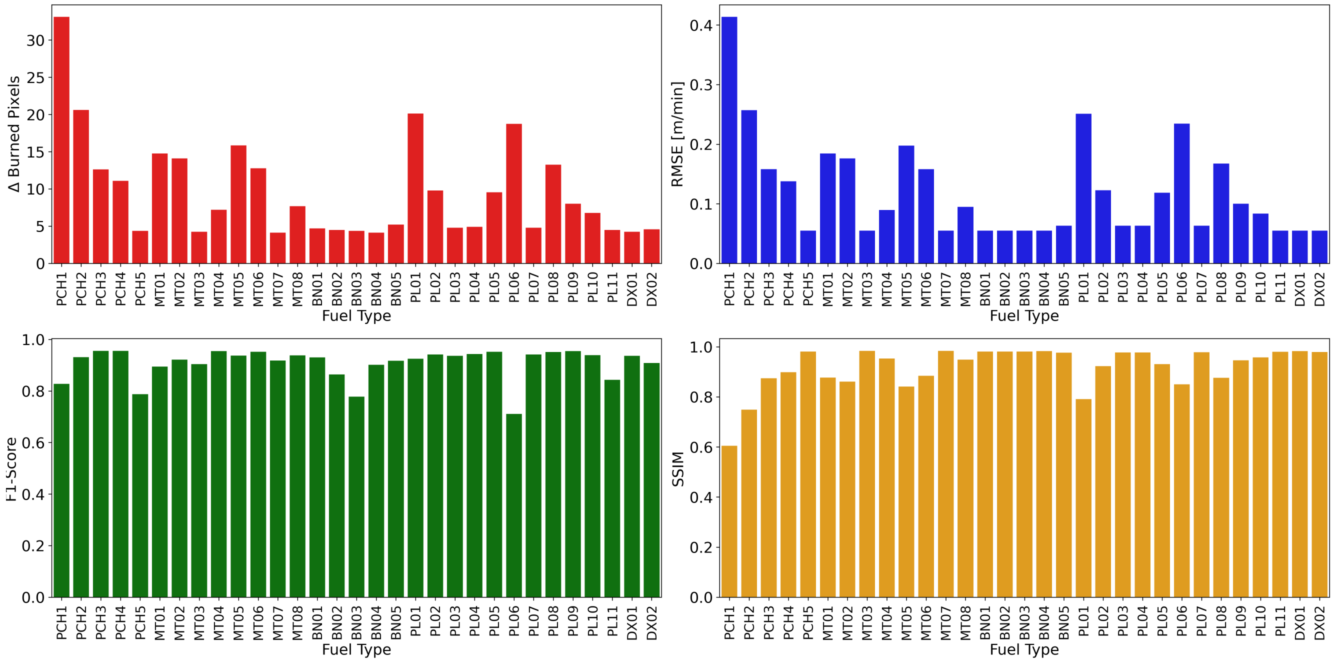

Supplementary Fig. S11. Full comparison of homogeneous fuels in the KITRAL system in order of fuels shown in Table S10 (Continued).

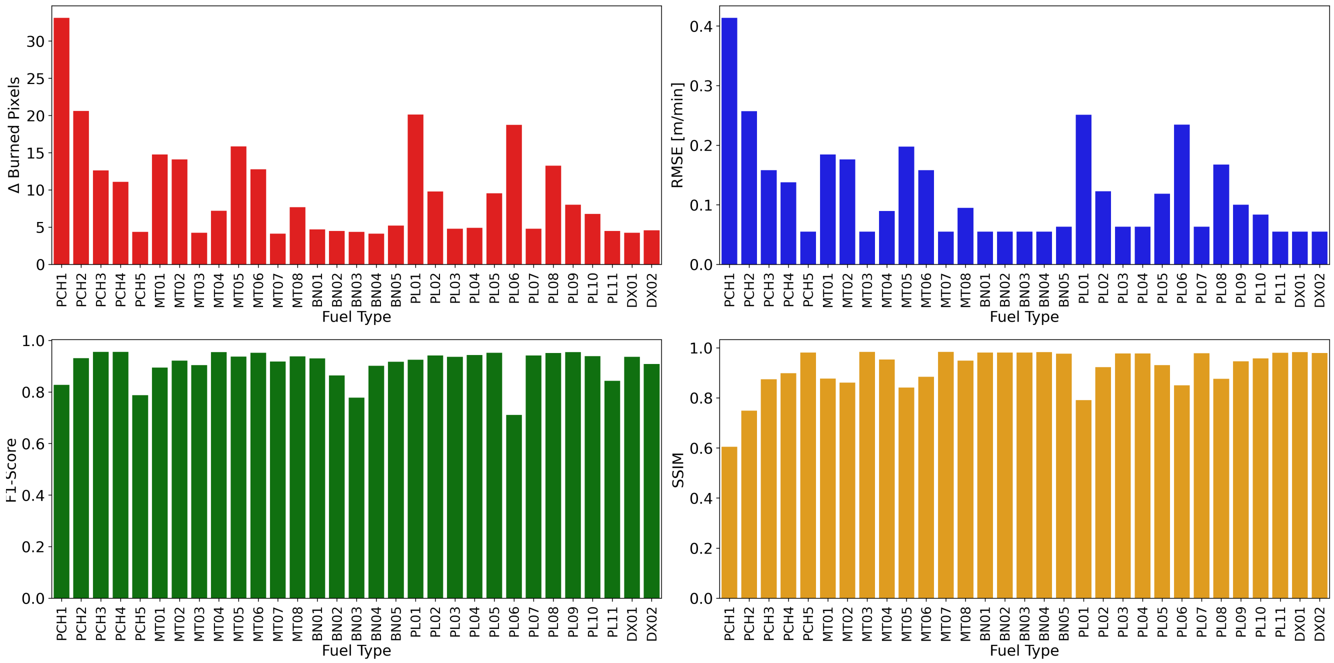

Supplementary Fig. S12. Accuracy and error metric plots for Cell2Fire fit on KITRAL (Chile).

Supplementary Table S10. Accuracy and error metric results from Cell2Fire fit on KITRAL (Chile), including computation time of simulation.

Instance	Δ Burned Cells	RMSE [m/min]	F1	SSIM	Time [s]
PCH1	33.11	0.41	0.83	0.61	0.47
PCH2	20.62	0.26	0.93	0.75	0.44
PCH3	12.61	0.16	0.96	0.87	0.27
PCH4	11.09	0.14	0.96	0.90	0.22
PCH5	4.36	0.05	0.79	0.98	0.08
MT01	14.77	0.18	0.90	0.88	0.22
MT02	14.11	0.18	0.92	0.86	0.22
MT03	4.24	0.05	0.90	0.98	0.07
MT04	7.21	0.09	0.96	0.95	0.15
MT05	15.84	0.20	0.94	0.84	0.27
MT06	12.77	0.16	0.95	0.88	0.26
MT07	4.12	0.05	0.92	0.98	0.06
MT08	7.68	0.09	0.94	0.95	0.14
BN01	4.69	0.05	0.93	0.98	0.09
BN02	4.47	0.05	0.86	0.98	0.08
BN03	4.36	0.05	0.78	0.98	0.06
BN04	4.12	0.05	0.90	0.98	0.07
BN05	5.20	0.06	0.92	0.98	0.10
PL01	20.13	0.25	0.93	0.79	0.32
PL02	9.80	0.12	0.94	0.92	0.17
PL03	4.80	0.06	0.94	0.98	0.09
PL04	4.90	0.06	0.94	0.98	0.10
PL05	9.54	0.12	0.95	0.93	0.19
PL06	18.74	0.23	0.71	0.85	0.12
PL07	4.80	0.06	0.94	0.98	0.09
PL08	13.27	0.17	0.95	0.88	0.24
PL09	8.00	0.10	0.96	0.95	0.17
PL10	6.78	0.08	0.94	0.96	0.13
PL11	4.47	0.05	0.84	0.98	0.06
DX01	4.24	0.05	0.94	0.98	0.09
DX02	4.58	0.05	0.91	0.98	0.09
**Average**	**9.66**	**0.15**	**0.91**	**0.92**	**0.17**

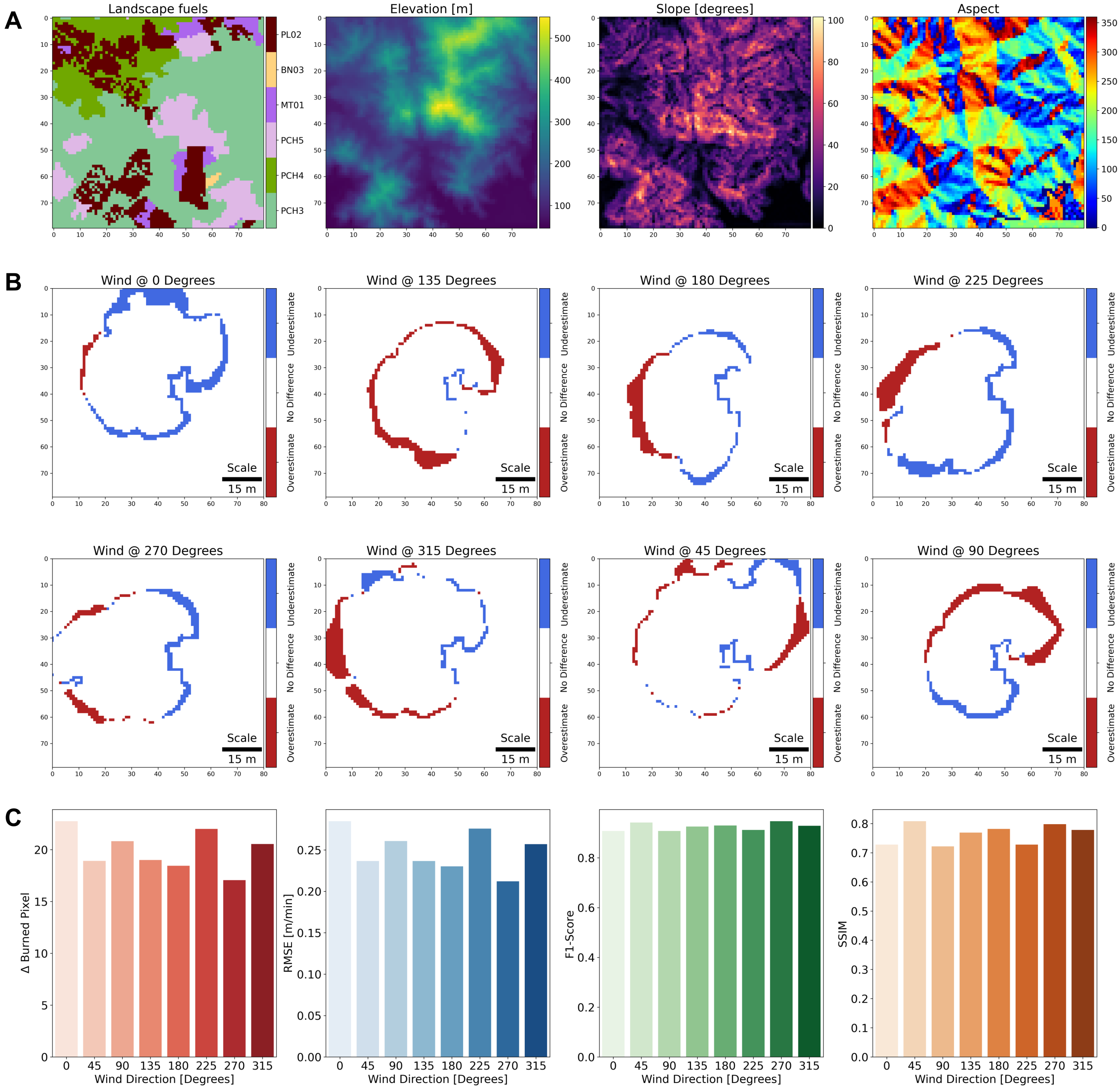

Supplementary Fig. S13. Fire spread simulation on a real landscape in Portezuelo, Chile. (A) Spatial distribution of fuel mapped based on KITRAL along with topographic information (elevation, slope, aspect) from a Digital elevation model (DEM) are shown. (B) Comparison of KITRAL and Cell2Fire-KITRAL's fire spread simulations at a constant wind speed (10 km/h) from for eight different wind directions (0°, 45°, 90°, 135°, 180°, 225°, 270°, 315°). In addition, the amount of overestimation of burned cells ($Area_{Cell2Fire} > Area_{FarSite}$) is highlighted in red, while underestimation ($Area_{Cell2Fire} < Area_{FarSite}$) is highlighted in blue. (C) Error (Δ Burned cells and RMSE) and accuracy metrics (F1-score and SSIM) for total burned cells in final simulations.

6 Uncertainty analysis

For the US real landscape, we first ran a grid search to find the influence of adjustment factors on HROS, BROS, FROS, and eccentricity on the simulation at each hour. In Cell2Fire, these four adjustment factors are applied at each cell to scale the elliptical propagation. We recorded accuracy and error metrics with respect to the reference FarSite burn scar. We used initial bounds of [0,3] and an interval size of 0.5 for the four ROS adjustment factors. Based on the initial findings, we ran another grid search using the following refined bounds:

- HROS Factor: [0.9, 1.1] with interval=0.1
- BROS Factor: [0.5, 1.5] with interval=0.1
- FROS Factor: [0.5, 1.5] with interval=0.1
- Eccentricity Factor [0.5, 1] with interval=0.1

To visualize the difference in results, we show three examples from the uncertainty analysis in Fig. S14 and their input parameters in Table S11.

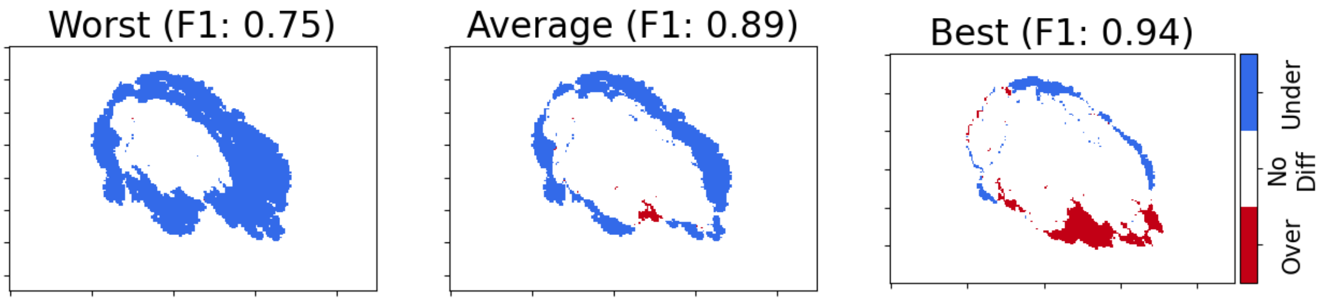

Supplementary Fig. S14. Comparison of worst, average, and best result by F1-score from the uncertainty analysis using Cell2Fire simulations on the US real landscape.

Case	HROS Factor	BROS Factor	FROS Factor	Eccentricity Factor	Fire period length	F1-score
Worst	0.9	1.0	1.0	0.9	0.5	0.7501
Average	0.9	1.1	0.8	0.7	2.0	0.8911
Best	1.1	1.4	1.1	0.8	2.0	0.9430

Supplementary Table S11. ROS adjustment factors and F1-scores for different cases in the uncertainty analysis.

For the Dogrib Fire (Canada), we created weather stream files by randomly adding noise (based on a range of values between 0 and 2) to wind speed, relative humidity, and temperature. We used constant parameters assuming severe fire weather conditions in accordance with the Canadian Forest Fire Weather Index (FWI) System (Duff Moisture Code (DMC): 64, Drought Code (DC): 535, Buildup Index (BUI): 99) (10). We also set hourly Fine Fuel Moisture Content (FFMC) between a range of 90 to 93 (10). To preserve some of the temporal trends, we used a window block of 3 hours. Ultimately, we created 1,000 weather stream files for the uncertainty analysis. We show the results in Fig. S15.

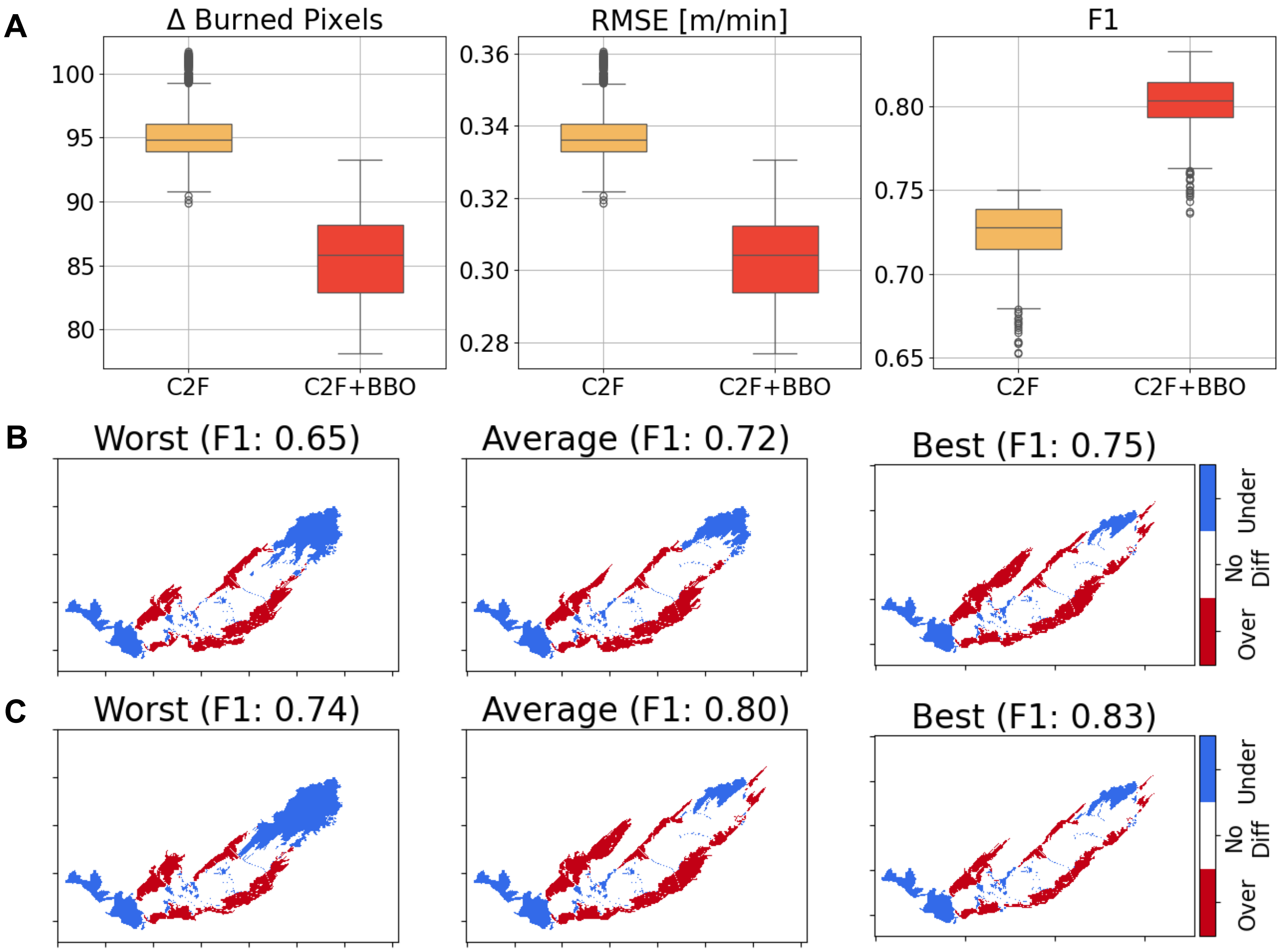

Supplementary Fig. S15. Uncertainty analysis results using weather data assuming severe fire weather conditions. (A) Comparison of evaluation metrics on Cell2Fire and Cell2Fire with BBO after simulating on all weather streams generated for the uncertainty analysis. (B) Comparison of selected examples by F1-score from **Cell2Fire** simulations in the uncertainty analysis. (C) Comparison of selected examples by F1-score from **Cell2Fire with BBO** simulations in the uncertainty analysis.

7 Sensitivity analysis

For the US real landscape, we used Spearman Correlation Coefficient (SPCC) to assess the relationship between the adjustment factors and the evaluation metrics. As shown in Table S12 We find that the eccentricity factor has the strongest relationship with the metrics ($SPCC_{RMSE}$=-0.5664 and $SPCC_{F1}$=0.7325). This strong influence is because eccentricity is a function of length-to-breadth ratio which is affected by wind speed. HROS factor has a weaker but statistically significant relationship ($SPCC_{RMSE}$=-0.0613 and $SPCC_{F1}$=0.1604). Fire period length also demonstrates a weaker but statistically significant relationship ($SPCC_{RMSE}$)=-0.1579 and $SPCC_{F1}$=0.0963). Here, fire period length is defined as the time duration for one simulation step in Cell2Fire (10). In contrast, BROS and FROS are both not statistically significant and recorded zero SPCC values.

Factor	Evaluation Metric	SPCC	SPCC p-value
HROS	RMSE	-0.0613	7.7882e-04
	F1	0.1604	9.812e-19
BROS	RMSE	0	1
	F1	0	1
FROS	RMSE	0	1
	F1	0	1
Eccentricity	RMSE	-0.5664	3.5098e-254
	F1	0.7325	0
Fire period length	RMSE	-0.1579	3.3369e-18
	F1	0.0963	1.2676e-07

Supplementary Table S12. SPCC and p-values for ROS adjustment factors and evaluation metrics.

To further explain the influence of input variables on fire spread, we created a ML-based surrogate model to compute ROS from the Rothermel (US) and FBP (Canada) equations. We trained XGBoost regression models using a dataset of input variables and outputs of HROS, BROS, and FROS computed via the Rothermel *R* library (8). We constrained the training dataset by wind speed and slope, which have been defined with lognormal and normal distributions in previous studies (14), and threshold the datasets to the 99th percentile to omit anomalies. We split the dataset into train and validation sets (80% and 20%) and cross-validate using Optuna to fine-tune optimal hyperparameters. We then extract a subset to analyze SHAPley summary plots and determine the influence of each input variable on model performance in Figs. S16 and S17. The XGBoost models for both the US and Canada were found to be highly accurate, as shown by the results in Table S13. We also show the training loss curves for both the US and Canada in Fig. S18 and Fig. S19, respectively.

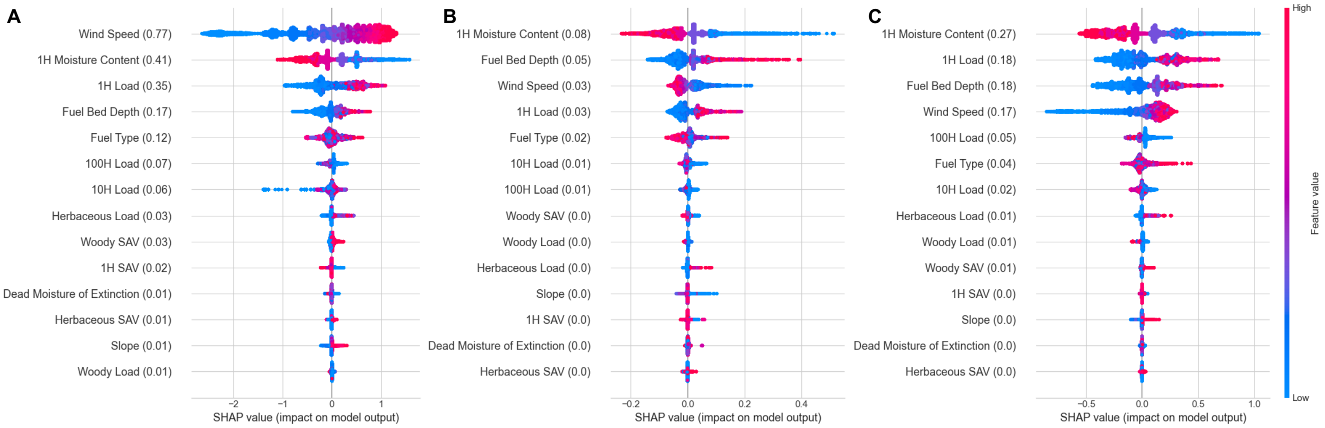

Supplementary Fig. S16. SHAP analysis of XGBoost trained on BehavePlus data. Input features and their SHAP values (in parentheses) displayed in descending order of impact on model predictions of (A) HROS, (B) BROS, and (C) FROS.

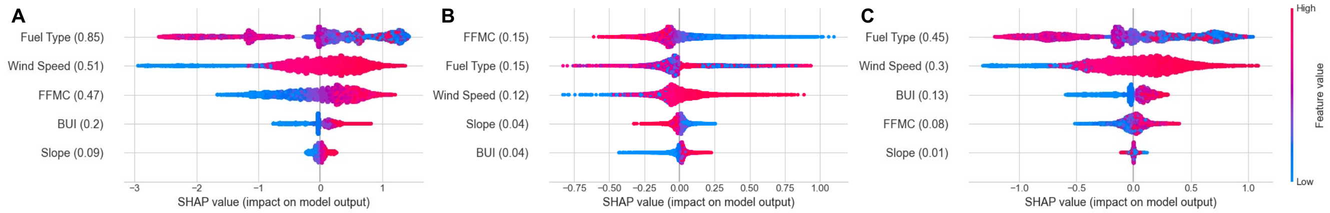

Supplementary Fig. S17. SHAP analysis of XGBoost trained on FBP data. Input features and their SHAP values (in parentheses) displayed in descending order of impact on model predictions of (A) HROS, (B) BROS, and (C) FROS.

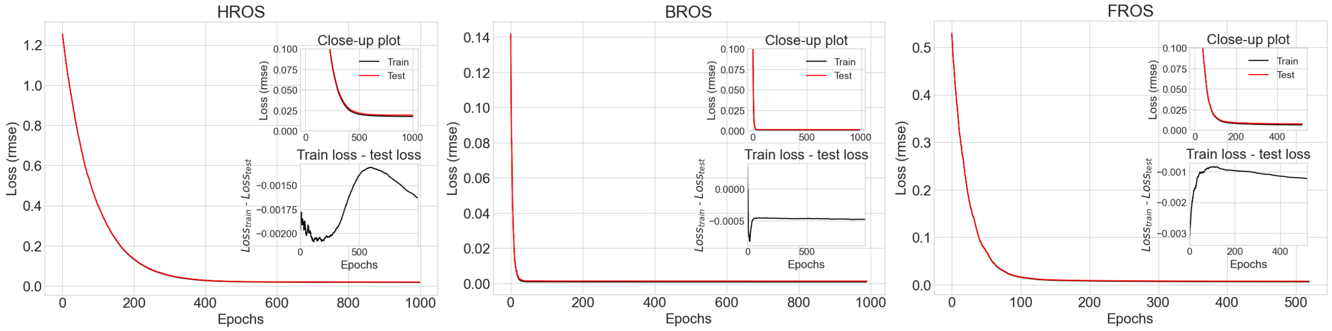

Supplementary Fig. S18. Training and test loss curves for the XGBoost model trained on BehavePlus data (US). Models were trained for a maximum of 1,000 epochs using hyperparameters found via cross-validation and optimization from Optuna.

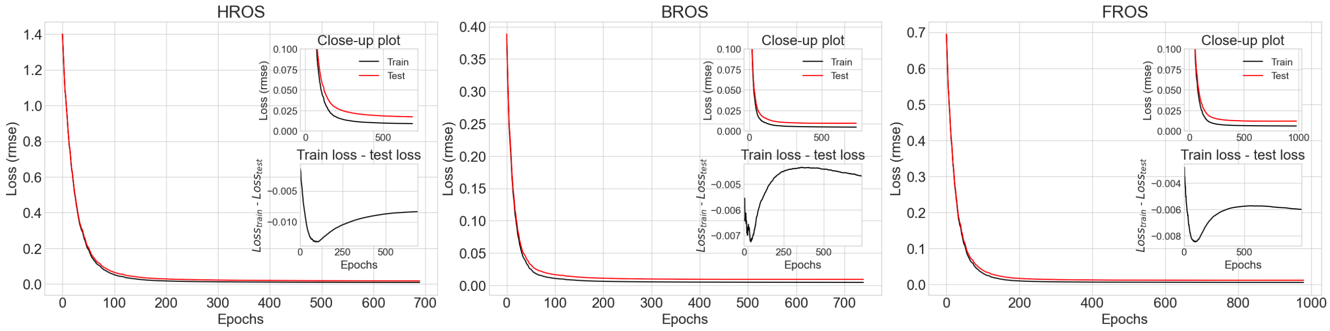

Supplementary Fig. S19. Training and test loss curves for the XGBoost model trained on FBP data (Canada). Models were trained for a maximum of 1,000 epochs using hyperparameters found via cross-validation and optimization from Optuna.

**Model**	**Test RMSE [m/min]**		
	**HROS**	**BROS**	**FROS**
US (BehavePlus)	0.0194	0.0013	0.0073
Canada (FBP)	0.0176	0.0090	0.0124

Supplementary Table S13. Comparison of test RMSE for HROS, BROS, and FROS in XGBoost models trained on data from BehavePlus (US) and FBP (Canada).

References

1. Scott, J. H. & Burgan, R. E. Standard fire behavior fuel models: a comprehensive set for use with Rothermel's surface fire spread model. Tech. Rep. Gen. Tech. Rep. RMRS-GTR-153, US Department of Agriculture, Forest Service, Rocky Mountain Research Station (2005).

2. Hirsch, K. G. *et al.* Canadian forest fire behavior prediction (FBP) system: user's guide. Tech. Rep. Special Report 7, Natural Resources Canada, Canadian Forest Service (1996).

3. Julio, G., Castillo, E. & Pedernera, P. Modelación de combustibles. *Actas del Taller Internacional sobre Progn. y Gestión en Incend. For.* 111–128 (1995).

4. Carrasco, J. *et al.* C2F-K: An open-source wildfire simulator based on Cell2Fire and the Chilean KITRAL system. *SSRN* (2023).

5. Sullivan, A. L. Wildland surface fire spread modelling, 1990–2007. 2: Empirical and quasi-empirical models. *Int. J. Wildland Fire* **18**, 369–386 (2009).

6. Papadopoulos, G. D. & Pavlidou, F.-N. A comparative review on wildfire simulators. *IEEE systems J.* **5**, 233–243 (2011).

7. Ghodrat, M., Shakeriaski, F., Fanaee, S. A. & Simeoni, A. Software-based simulations of wildfire spread and wind-fire interaction. *Fire* **6**, 12 (2022).

8. Vacchiano, G. & Ascoli, D. An implementation of the Rothermel fire spread model in the R programming language. *Fire Technol.* **51**, 523–535 (2015).

9. Alexander, M. *et al.* Estimating the length-to-breadth ratio of elliptical forest fire patterns. In *Proceedings of the eighth conference on fire and forest meteorology*, vol. 29, 85–04 (Society of American Foresters, Detroit, MI, USA, 1985).

10. Pais, C., Carrasco, J., Martell, D. L., Weintraub, A. & Woodruff, D. L. Cell2fire: A cell-based forest fire growth model to support strategic landscape management planning. *Front. For. Glob. Chang.* **4**, 692706 (2021).

11. Finney, M. A. Farsite: fire area simulator–model development and evaluation. Tech. Rep. Tech. Rep. RMRS-RP-4, U.S. Department of Agriculture, Forest Service, Rocky Mountain Forest and Range Experiment Station, Ogden, Utah, USA (1998).

12. Anderson, H. E. Predicting wind-driven wind land fire size and shape. Tech. Rep. INT-305, US Department of Agriculture, Forest Service, Intermountain Forest and Range Experiment Station (1983).

13. Ghisu, T., Arca, B., Pellizzaro, G. & Duce, P. An optimal cellular automata algorithm for simulating wildfire spread. *Environ. Model. & Softw.* **71**, 1–14 (2015).

14. Plischke, E., Rabitti, G. & Borgonovo, E. Computing shapley effects for sensitivity analysis. *SIAM/ASA J. on Uncertain. Quantification* **9**, 1411–1437 (2021).
